# Landscape Context and Environmental Stochasticity Shape Genetic Management Outcomes in an Amphibian

**DOI:** 10.1111/eva.70310

**Published:** 2026-08-05

**Authors:** Hanne Haugen, Børre K. Dervo, Arne Linløkken, Jan Heggenes, Kjartan Østbye

**Affiliations:** ^1^ Department of Forestry and Wildlife Management Inland Norway University of Applied Sciences, Campus Evenstad Inlandet Norway; ^2^ Norwegian Institute for Nature Research (NINA) Oslo Norway; ^3^ Department of Natural Sciences and Environmental Health University of South‐Eastern Norway, Campus Bø Notodden Norway; ^4^ Department of Biosciences Center for Ecological and Evolutionary Synthesis (CEES), University of Oslo Oslo Norway

**Keywords:** amphibian, environmental stochasticity, genetic diversity, landscape context, population viability analysis (PVA), *Triturus cristatus*

## Abstract

Landscape context can influence demographic rates and, consequently, the population genetic processes determining levels of genetic diversity. However, the role of landscape context is not always sufficiently considered in conservation management. For species with complex life cycles, such as amphibians, landscape context is particularly relevant because they rely on both aquatic and terrestrial habitats, the quality of which can vary across landscapes. Understanding how landscape context may influence these genetic processes is therefore important for effective management. We used population viability analysis (PVA) to simulate how landscape context may influence genetic and demographic outcomes for the great crested newt ( *Triturus cristatus* ) using two landscape‐specific models representing boreal forest and agricultural landscapes. We assessed the sensitivity of model outcomes to parameter perturbations and evaluated management scenarios and potential threats at the metapopulation level. A key result was that environmental stochasticity strongly influenced genetic dynamics in both landscapes. The low‐stochasticity forest model retained genetic diversity, responded strongly to management actions, and relied less on gene flow. In contrast, the high‐stochasticity agricultural model experienced stronger genetic drift, responded weakly to management actions, and relied more on continuous gene flow. Our findings suggest that enhancing gene flow alone may be insufficient for maintaining genetic diversity, and that improving resilience to environmental stochasticity should be considered an important part of genetic management.

## 1 Introduction

Understanding how local population dynamics influence genetic and demographic processes is important for effective management. These dynamics are affected by ecological factors operating at multiple spatial scales, from fine‐scale habitat characteristics to large‐scale factors such as climate, geology, and anthropogenic pressure (Burrow and Maerz 2022; Caruso and Rissler 2019; Shemesh et al. 2022). When focusing on local population dynamics, the large‐scale factors may be easily overlooked. These factors, however, may give rise to fundamentally different population dynamics depending on landscape context (Cayuela et al. 2022; Hardy et al. 2023). Hence, understanding the role of landscape context may be essential for developing effective species‐level management strategies.

Landscape context may be particularly relevant for species with complex life cycles. Many amphibians rely on both aquatic and terrestrial habitats during their life cycle, and the extent and quality of these habitats may vary between landscapes, sometimes in opposite directions. For instance, nutrient‐rich agricultural ponds can benefit amphibian larvae by enhancing survival until metamorphosis (Rowland et al. 2016; Youngquist and Boone 2021), while surrounding terrestrial habitat affected by fragmentation and degradation may reduce adult survival (Băncilă et al. 2023; Porej et al. 2004). In contrast, breeding ponds in nutrient‐poor forests may represent suboptimal larval habitats, but the surrounding forest can offer larger, more continuous high‐quality terrestrial habitat when anthropogenic pressure is low (Rannap et al. 2012).

Amphibians are undergoing global declines and are one of the most vulnerable vertebrate groups (Chanson et al. 2023). Population viability analysis (PVA) is an important tool for developing conservation strategies for vulnerable species (Beissinger and Westphal 1998). It is typically used to simulate population dynamics over time, assess population extinction risk under various future or management scenarios, and identify key demographic factors that disproportionately affect extinction risk (Akçakaya and Sjögren‐Gulve 2000). Although the number of PVA studies in amphibians is increasing, landscape context is rarely explicitly considered.

Modern PVA software often allows for the integration of genetic data, but genetic diversity is seldom treated as an explicit model outcome (but see Auffarth et al. (2017)). This is also the case for amphibian PVA studies, even for well‐studied species such as *Triturus cristatus* (Griffiths and Williams 2000; Karlsson et al. 2007). Genetic diversity is a key component of biodiversity, important for individual fitness and a population's adaptive potential (Hoffmann et al. 2017; Keller and Waller 2002), and recognized as key for amphibian conservation (Pabijan et al. 2020). Including genetic metrics as model outcomes could therefore strengthen the relevance of amphibian PVA studies for management, as genetic and demographic metrics may respond differently to environmental threats and management interventions.

In this study, we use the great crested newt ( *Triturus cristatus* ), a pond‐breeding amphibian listed as Least Concern globally (IUCN 2023), but regionally classified as threatened or otherwise of conservation concern in many parts of its range (Dufresnes and Perrin 2015), as a model species to assess how landscape context influences population viability, with a particular emphasis on genetic outcomes. We construct two alternative PVA models representing populations in two contrasting landscapes: a boreal forest system characterized by low larval productivity, but mostly continuous terrestrial habitat, and an agricultural system with high larval productivity but more disturbed and fragmented terrestrial habitat.

We evaluate genetic diversity, effective population size, and census population size, and assess their sensitivity to parameter changes. For the baseline models, we also examine the distribution of effective‐to‐adult census population size ratios (*N*_e_/*N*) with and without environmental stochasticity. We further extend the models to metapopulations, incorporating landscape‐modulated dispersal rates and spatial variation in local habitat conditions, to assess how different management strategies and potential threats influence genetic diversity, genetic differentiation, and census population size across landscape contexts.

## 2 Material and Methods

### 2.1 Study Areas

**FIGURE 1:**
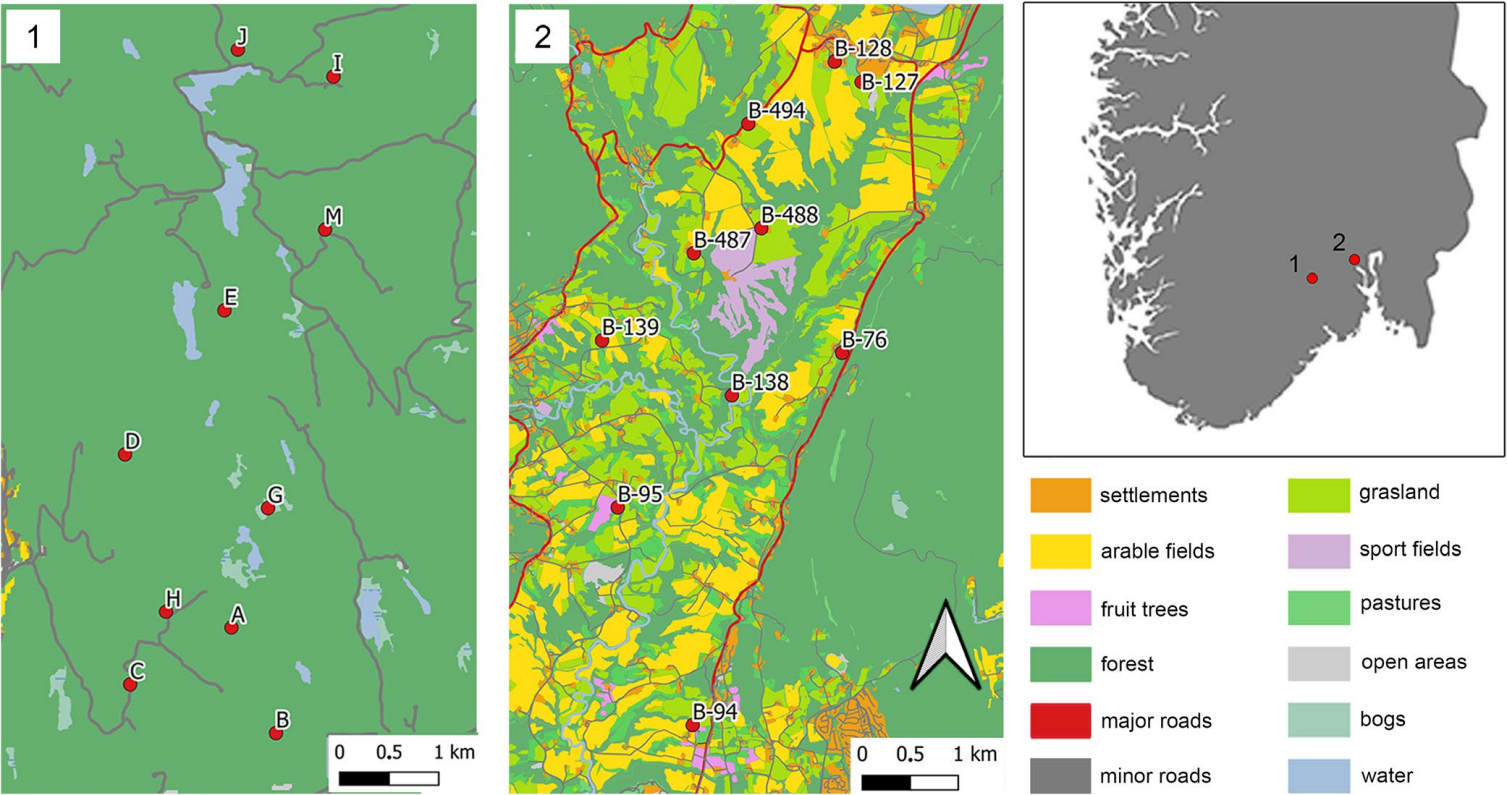
Overview of the forested (1) and agricultural (2) landscapes. Great crested newt populations are shown as red points, with population IDs indicated by letters/numbers. The background represents land cover.

Genetic and environmental data were collected from a previous study conducted in southeastern Norway (Haugen et al. 2023). The forest populations are located within a boreal forest ecosystem (59°37′34.9″N, 9°19′15.4″E; mean elevation = 371 m.a.s.l.; Figure 1). The breeding ponds are dystrophic, nutrient‐poor (mean Tot *N* = 544 μg/L, mean Tot *p* = 18 μg/L), with low aquatic vegetation cover. Average nearest‐neighbor distance between ponds is 998 m (SD = 307 m). The surrounding forest is dominated by pine ( *Pinus sylvestris* ) and spruce ( *Picea abies* ) growing on glacial moraine deposits. About 40% of the surrounding forested landscape is near‐natural forest, while the remainder is affected by intensive forestry practices, including clear‐cuts and gravel roads.

The agricultural populations inhabit a highly modified agricultural landscape (59°50′53.1″N, 10°14′16.5″E; mean elevation = 103 m.a.s.l.; Figure 1) with nutrient‐rich breeding ponds (mean Tot *N* = 1484 μg/L, mean Tot *p* = 101 μg/L) and abundant aquatic vegetation, but also more fragmented and degraded terrestrial habitats. Average nearest‐neighbor distance between ponds is 1248 m (SD = 665 m). The majority of these ponds are man‐made, except for one small oxbow pond (B‐138, Figure 1). The surrounding landscape consists of arable fields, pastures, urban development and forest patches dominated by broad‐leaved trees.

### 2.2 Population Viability Analysis

The population viability analyses (PVA) were performed with the software Vortex v.10.6.0.0 (Lacy and Pollak 2023), a widely used PVA software that simulates deterministic and stochastic processes, and models the effects of demographic factors such as fecundity, mortality and dispersal. By accounting for environmental and demographic stochasticity and genetic drift, users may assess long‐term population viability, including changes in population size and heterozygosity (Lacy et al. 2024).

### 2.3 Model Parameterization

The forest and agricultural models were parameterized based on literature, expert knowledge, and general knowledge of the species ecology, including how differences in habitats may influence vital rates (Table 1, Tables S1 and S2). Great crested newt populations have been shown to have contrasting population dynamics, ranging from low recruitment combined with low, stable adult mortality to high recruitment combined with higher, less stable adult mortality (Cayuela et al. 2020). We assumed that the contrasting habitat characteristics of the forest and agricultural study areas would give rise to similar contrasts in population dynamics (Table 1). Nutrient‐rich agricultural ponds with abundant aquatic vegetation were assumed to support higher fecundity than dystrophic forest ponds with sparser vegetation, due to higher larval productivity and greater availability of egg‐laying substrate (Gustafson et al. 2009; Sstatecsny et al. 2004). Conversely, fragmented and degraded terrestrial habitats in agricultural landscapes were assumed to result in higher and more variable adult mortality compared to the larger and more continuous terrestrial habitats in forest, due to more exposure to desiccation and potentially lower availability of vital resources, such as suitable overwintering sites (Langton et al. 2001; Rannap et al. 2012). Juvenile mortality was assumed to be the same in both models and decreasing with age (Cayuela et al. 2017).

**TABLE 1:** PVA model input parameters for the forest and agricultural models (left) and parameter perturbation ranges in sensitivity analyses 1 and 2 (right).

Parameters	Models		Sensitivity analyses	
	Forest	Agricultural	1	2
Reproductive system	Polygamous			
Extinction definition	Only 1 sex remains			
Lethal equivalents	3.14			
Maximum lifespan (years)	18			
Maximum age of reproduction (years)	17			
Dispersal age (years)	1–3			
Dispersal sex	Both			
Sex ratio at birth	1:1			
% females breeding	Density‐dependent based on aquatic vegetation cover			
% males in breeding pool	25.51			
Age at maturation (years)	F 4, M 4	F 4, M 3		
Fecundity ± SD (no. metamorphs per female)	5.02 ± 2.5 (SD)	5–11 ± 2.5 (SD)	±5	0–20 (SD: 0–10)
Mortality (%)				
Juveniles (age 1) ± SD	68 ± 23 (SD)	68 ± 23 (SD)	±5	16–81 (SD: 0–24)
Juveniles (age 2) ± SD	54 ± 24 (SD)	54 ± 24 (SD)	±5	16–81 (SD: 0–24)
Juveniles (age 3) ± SD	38 ± 23 (SD)	38 ± 23 (SD)	±5	16–81 (SD: 0–24)
Juveniles (age 4) ± SD	27 ± 14 (SD)	30 ± 20 (SD)	±5	16–81 (SD: 0–24)
Adults ± SD	15 ± 5 (SD)	30 ± 20 (SD)	±5	13–74 (SD: 0–24)
Carrying capacity (no. juveniles and adults)	15,120	8714	±5	1–46,430
Aquatic vegetation cover (m^2^)	744 (372)^a^	600	±5	1–3260

*Note:* Mortality rates are given as percentages (%) and fecundity as number of metamorphs per female per brood. SD denotes the standard deviation for each parameter. For mortality rates, it represents environmental variation, and for fecundity, variation in offspring number per female. Fecundity, estimated from egg and larval survival, was modeled as density‐dependent in the agricultural model (see Supporting Information for details).

^a^ Because vegetation cover was less dense in forest ponds, we assumed that each female required twice as much area to lay her eggs compared to agricultural ponds. Accordingly, forest models were run with half the vegetation cover (744/2 = 372).

Fecundity, the number of metamorphs per female per brood, was drawn from a normal distribution (Table 1). Based on reported clutch size and egg/larvae mortality in the great crested newt (Arntzen and Teunis 1993; Cvijanović et al. 2009; Oldham 1994), we set fecundity to five metamorphs per female. This yielded a stable deterministic growth rate (*λ* = 1) in the forest model. In the agricultural model, fecundity was modeled as density‐dependent (range 5–11), reflecting the higher larval productivity and greater likelihood of density‐regulated larval mortality (details in Supporting Information). The proportion of breeding females was modeled as density‐dependent and determined by the availability of suitable egg‐laying substrate, that is, aquatic vegetation cover (details in Supporting Information), assuming that the contribution of females without access to such substrate was negligible (Miaud 1994). Age at maturation was set to 4 years in both models, except for males in the agricultural model, for which it was set to 3 years. This was to reflect the geographic variation in age at maturation observed in Norwegian great crested newt populations (Dolmen 1983). The number of lethal equivalents, that is, the effect of inbreeding depression on first‐year survival, was set to 3.14 (Ralls et al. 1988).

Environmental stochasticity (ES) was modeled in Vortex by drawing annual mortality rates from binomial distributions defined by the specified mean mortality rates and their associated standard deviations (Table 1). Correlation in ES among subpopulations was set to 0.5 (Cayuela et al. 2020).

### 2.4 Sensitivity Analyses

The forest and agricultural models were used to assess the sensitivity of heterozygosity (*H*_e_), effective population size (*N*_e_), and total census population size (*N*_T_; juveniles and adults) to changes in model input parameters. The initial population size was set to 500, with no dispersal. One parameter at a time was varied (with others constant), including fecundity and its SD, juvenile and adult mortality, and SD for mortality (representing environmental stochasticity), carrying capacity, and aquatic vegetation cover determining the percentage of breeding females (Table 1).

Sensitivity analysis is traditionally conducted by changing parameters by a fixed value. However, since parameters vary in their natural variability and in how amenable they are to management (Manlik et al. 2018), we also performed a second sensitivity analysis by varying input parameters based on realistic ranges.

In sensitivity analysis 1, each parameter was adjusted by ±5 units (i.e., absolute changes in parameter values), and linear regressions fitted to estimate the slope for each of the outcome variables (Table 1). In sensitivity analysis 2, each parameter was changed within realistic min‐max ranges derived from literature and field data, and by 30 incremental steps (Table 1). We calculated the differences in model outcomes by subtracting the results of the simulations using the minimum or maximum input values from those using the baseline parameters, and ranked the parameters based on the largest observed differences.

Each simulation was run for 50 years with 1000 iterations. Mean outcomes after 50 years were calculated across all simulations, including those in which extinction occurred. In these cases, outcome values were set to zero. Alternatively, means could have been calculated using only extant populations; however, this would have introduced survival bias and would not reflect the full range of possible population trajectories.

The mean *H*_e_ and *N*_T_ at year 50 were extracted directly from Vortex. In Vortex, heterozygosity is estimated from changes in allele frequencies over time. Effective population size (*N*_e_) was estimated from the mean proportional change in *H*_e_ from year 0 to 50, adjusted for the number of generations, following Holleley et al. (2014); equation provided in Supporting Information.

### 2.5 The Baseline Models and *N*_e_/*N*

We estimated the effective‐to‐adult census population size ratio (*N*_e_/*N*) for each iteration for three versions of both the forest and agricultural models: the baseline models, baseline models with environmental stochasticity in adult mortality exchanged between landscapes, and models without environmental stochasticity at any life stages. For each iteration, the harmonic mean of the adult census population size (*N*) was calculated. Because *N*_e_ and *N* must be estimated for matching time intervals (Waples 2005), *N*_e_ was calculated for the period from year 0 + t to year 50, while the harmonic mean of *N* was calculated from year 0 to year 50‐t, where t represents generation time. The resulting *N*_e_/*N* values from 1000 iterations were visualized as density plots to assess their distribution and modal values.

### 2.6 Metapopulation Simulations

The forest and agricultural models were expanded into metapopulations by incorporating genetic data from 10 spatially adjacent populations in each landscape, and by including dispersal among them. Initial population sizes were estimated based on catch per unit effort data (Table S2).

Dispersal rates between populations were weighted by the cost of moving through the intervening landscape. Cost surfaces were generated from landscape models we created by using remote sensing data and natural resources maps (details in Supporting Information). High costs were assigned to areas with dry microclimate and gravel roads in the forest landscape, and rivers, major roads, pastures, and unvegetated areas and cultivated fields in the agricultural landscape (Haugen et al. 2023). In pond‐breeding amphibians, juveniles are generally considered the primary dispersal stage (Semlitsch 2008), a pattern also observed for *Triturus cristatus* (Kupfer and Kneitz 2000; Müllner 2001). Hence, dispersal was limited to juveniles in our models.

Terrestrial carrying capacity was estimated based on habitat area within a 300 m radius around each pond and the maximum observed density (details in Supporting Information). In the forest landscape, suitable habitat was defined as moist or mesic forests, excluding areas with low field vegetation cover (Vuorio et al. 2015). In the agricultural landscape, suitable habitats included forests and uncultivated fields (Rannap et al. 2009). Temporal changes in carrying capacity were estimated from aerial photos taken over the last 20 years. We found an annual decline of 0.3% in both landscapes, caused by forestry and land‐use conversion.

Stochastic catastrophic events were modeled for drought‐prone ponds, which were only found in the forest landscape. Historical precipitation data indicates that droughts occur with a frequency of 14% (Norsk_KlimaServiceSenter 2024). Droughts were assumed to cause complete reproductive failure in shallow ponds (< 50 cm) and 50% failure in moderately shallow ponds (50–100 cm).

In the corridor enhancement scenario (Table 2), we modified the forest landscape to reflect a gentler harvest regime with continuous canopy cover. In the agricultural landscape, we added 30 m wide dispersal corridors. Corridor enhancements led to median dispersal rates tripling in the forest model (from 0.07 to 0.21), while the change in the agricultural model was more modest, increasing from 0.18 to 0.24 (Table S3).

**TABLE 2:** Metapopulation scenarios with abbreviations and descriptions.

ABBR.	Scenarios	Description
BASE	Baseline	
COR	Corridor enhancement	In forest, a gentle harvest regime with continuous canopy cover reducing dry areas (barriers). In agricultural landscape, 30 m wide dispersal corridors.
HAB	Habitat enhancement	In forest, a gentle harvest regime with continuous canopy cover, increasing areas with beneficial microclimatic conditions. In agricultural landscape, adding suitable habitat within 100 m radius around breeding pond.
ADES20, ADES5	Environmental stochasticity in adult mortality	In forest, increased environmental stochasticity in adult mortality from 5 to 20 (ADES 20). In agricultural landscape, reduced environmental stochasticity in adult mortality from 20 to 5 (ADES5).
JUV1ES	Environmental stochasticity in juvenile mortality	In both landscapes, reduced environmental stochasticity for first‐year juveniles from 23 to 10.
JUV1	Juvenile mortality	In both landscapes, reduced first‐year juvenile mortality in from 68% to 58%.
ADM	Adult mortality	In agricultural landscape, reduced adult mortality from 30% to 20%.
FEC	Fecundity	In forest, increased fecundity from 5.02 to 6.02. In agricultural landscape, increased intercept in density‐dependent function for fecundity from 10.95 to 11.95.
0MIG	Zero migration	All dispersal rates set to zero.
DRY	Pond droughts	Extreme drought (1 per 50 years) leading to complete reproduction failure in all ponds.
TER	Terrestrial catastrophes	Terrestrial catastrophes (1 per 50 years) leading to 50% proportional increase in mortality for all post‐metamorphic life stages.
ADES5 + ADM	Combined management actions	In agricultural landscape, reduced environmental stochasticity in adult mortality (ADES5) combined with one of the following scenarios: ADM, JUV1, COR, HAB, FEC.
ADES5 + JUV1		
ADES5 + COR		
ADES5 + HAB		
ADES5 + FEC		

*Note:* Changes in environmental stochasticity were implemented by adjusting the standard deviation of the probability distribution from which annual mortality rates were drawn.

In the habitat enhancement scenario for the forest metapopulation (Table 2), we created landscape models reflecting microclimatic conditions and field vegetation cover under a gentler harvest regime with continuous canopy cover (details in Supporting Information). For the agricultural metapopulation, habitat enhancements consisted of adding a 100 m radius of suitable habitat around each breeding pond. Habitat enhancements slightly reduced median carrying capacity in the forest (17,742 to 17,012), likely due to reduced field‐vegetation cover, but seven out of 10 forest subpopulations experienced an increase. The agricultural metapopulation showed a substantial increase in carrying capacity (13,027 to 18,579).

Additionally, two scenarios representing catastrophic events were included: one representing extreme droughts affecting all ponds, and another representing terrestrial catastrophes affecting juveniles and adults (Table 2).

Based on the findings in the sensitivity analyses we formulated additional metapopulation scenarios, both representing management interventions and potential threats (Table 2). Each model was simulated in Vortex for 50 years using 10,000 iterations, and mean H_e_, *N*_T_, and pairwise genetic differentiation (G_ST_) at year 50 were extracted. As in the sensitivity analyses, we included both extant and extinct populations when calculating the mean across iterations.

## 3 Results

### 3.1 Sensitivity Analyses

In the sensitivity analyses, the most notable difference between the forest and the agricultural models was in the magnitude of change in the effective population size *N*_e_ (Figure S1). This difference was most evident when juvenile mortality was reduced or fecundity increased. In contrast, changes in census population size *N*_T_ were in most cases much more similar across landscape context (Figure S2). Differences in heterozygosity were also modest, which is expected given the limited potential for heterozygosity to increase in closed populations (Figure S3).

**FIGURE 2:**
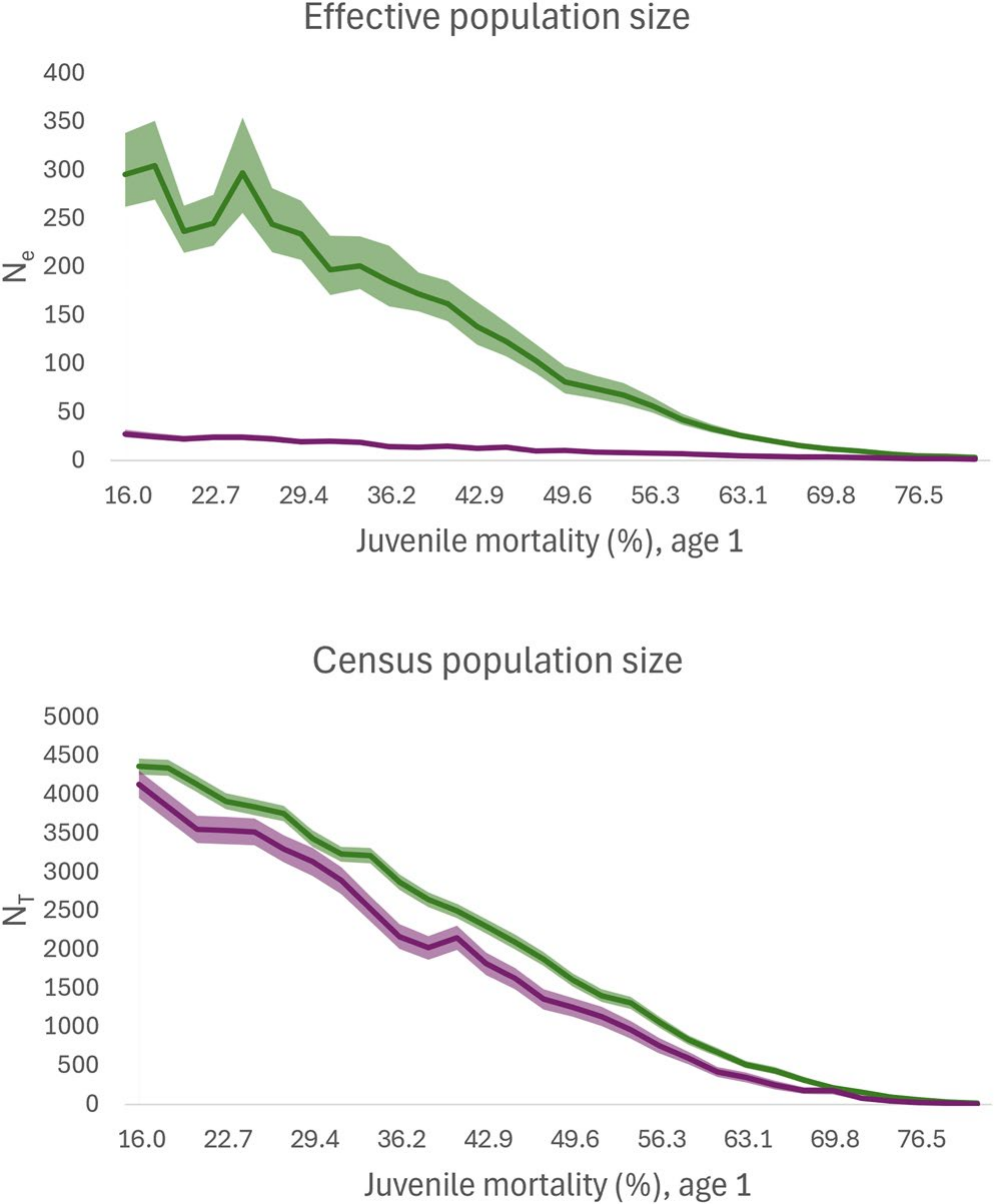
Results from sensitivity analysis 2. Green lines represent the forest model, and purple lines represent the agricultural model. The figure shows how effective population size (*N*_e_; upper panel) and census population size (*N*_T_; lower panel) change with increasing juvenile mortality at age 1. Shaded confidence bands indicate ±1.96 × SE.

The observed pattern can be illustrated by an example from sensitivity analysis 2. Here, reduced juvenile mortality (age 1) increased *N*_e_ about 12 times more in the forest model than in the agricultural model (Table 3, Figure 2). The corresponding difference for *N*_T_ was only 0.028 (Table 3, Figure 2).

**TABLE 3:** Results from sensitivity analysis 2.

Rank	Parameters	Forest	Parameters	Agricultural
		*H*_e_		*H*_e_
1	Juvenile mortality (age 1)	0.109	Juvenile mortality (age 1)	0.309
2	Fecundity	0.103	Adult mortality	0.258
3	Juvenile mortality (age 2)	0.093	Fecundity	0.213
4	Juvenile mortality (age 3)	0.078	Juvenile mortality (age 2)	0.210
5	ES in Juvenile mortality (age 1)	0.072	ES in adult mortality	0.202
		*N*_e_		*N*_e_
1	Fecundity	290.0	Juvenile mortality (age 1)	24.0
2	Juvenile mortality (age 1)	282.5	Adult mortality	9.0
3	Juvenile mortality (age 2)	161.7	Juvenile mortality (age 2)	8.4
4	Juvenile mortality (age 3)	35.5	ES in adult mortality	7.2
5	ES in Juvenile mortality (age 1)	34.7	ES in Juvenile mortality (age 1)	6.1
		*N*_T_		*N*_T_
1	Fecundity	6094	Juvenile mortality (age 1)	3980
2	Juvenile mortality (age 1)	4093	Adult mortality	1511
3	Juvenile mortality (age 2)	1837	Juvenile mortality (age 2)	1413
4	Juvenile mortality (age 3)	662	Fecundity	1182
5	Juvenile mortality (age 4)	248	Juvenile mortality (age 3)	444
↓	Juvenile mortality (age 3)	*N*_T_ = 0	Juvenile mortality (age 3)	*N*_T_ = 0
↓	Juvenile mortality (age 4)	*N*_T_ = 0	Juvenile mortality (age 4)	*N*_T_ = 0
↓	Aquatic vegetation	*N*_T_ = 0	Aquatic vegetation	*N*_T_ = 0
↓	Fecundity	*N*_T_ = 0	Fecundity	*N*_T_ = 0
↓	Carrying capacity	*N*_T_ = 0	Carrying capacity	*N*_T_ = 0
↓	Adult mortality	*N*_T_ = 0	Adult mortality	*N*_T_ = 0

*Note:* Input parameters are ranked by the greatest increase in model outcomes (*H*_e_, *N*_e_, *N*_T_) relative to the baseline model, when each parameter is set to its minimum or maximum value. Downward arrows indicate lowest ranking parameters (*N*_T_ = 0).

This pattern persisted also if populations that became extinct during the simulations were excluded, although the differences were less pronounced. In this case, the forest model had approximately three times higher *N*_e_ than the agricultural model.

The greater importance of environmental stochasticity for genetic outcomes was evident in both sensitivity analyses. Input parameters representing environmental stochasticity (Table 1) were consistently among the top five ranked variables in both models when comparing genetic outcomes, but never for demographic outcomes (Table 3, Tables S4 and S5). Environmental stochasticity in adult mortality was the most influential stochastic parameter in sensitivity analysis 1 for both models. In sensitivity analysis 2, however, environmental stochasticity in juvenile mortality (age 1) was more influential in the forest model. This likely reflects the already low baseline stochasticity in adult mortality in the forest model, leaving little to be gained from further reductions.

In the sensitivity analyses, fecundity and mortality rates were consistently ranked high. This was true for both landscape models and for both genetic and demographic outcomes. However, there were some differences in ranking order. In sensitivity analysis 1, fecundity, followed by adult mortality, consistently ranked as the most important input parameters in both landscape models (Table S4). In sensitivity analysis 2, however, fecundity appeared more influential in the forest than in the agricultural model, especially for *N*_e_ (Table 3, Table S5). Conversely, adult mortality was clearly more important in the agricultural than in the forest model for all outcomes in sensitivity analysis 2.

In both landscape models, changes in mortality of younger juveniles had stronger positive effects (increases in outcome variables) than changes in mortality of older juveniles. In contrast, for negative outcomes (population collapse), changes in mortality of older juveniles had a stronger effect. Population collapses also followed from high adult mortality, low fecundity, and extremely low carrying capacity and aquatic vegetation cover (Table 3, Table S5).

### 3.2 The Baseline Models and *N*_e_/*N*

**FIGURE 3:**
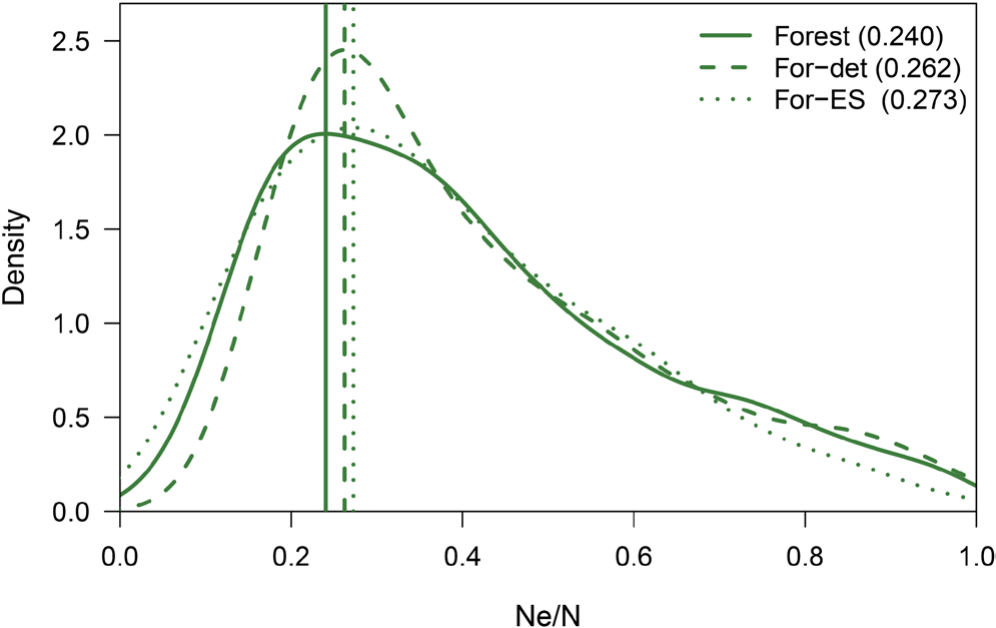
The distribution of *N*_e_/*N* values for 0 < *N*_e_/*N* ≤ 1 in the forest baseline model (Forest), the forest model without environmental stochasticity (For‐det), and the forest model with adult environmental stochasticity switched with the agricultural baseline model. Vertical lines indicate the modal values (density peaks).

**FIGURE 4:**
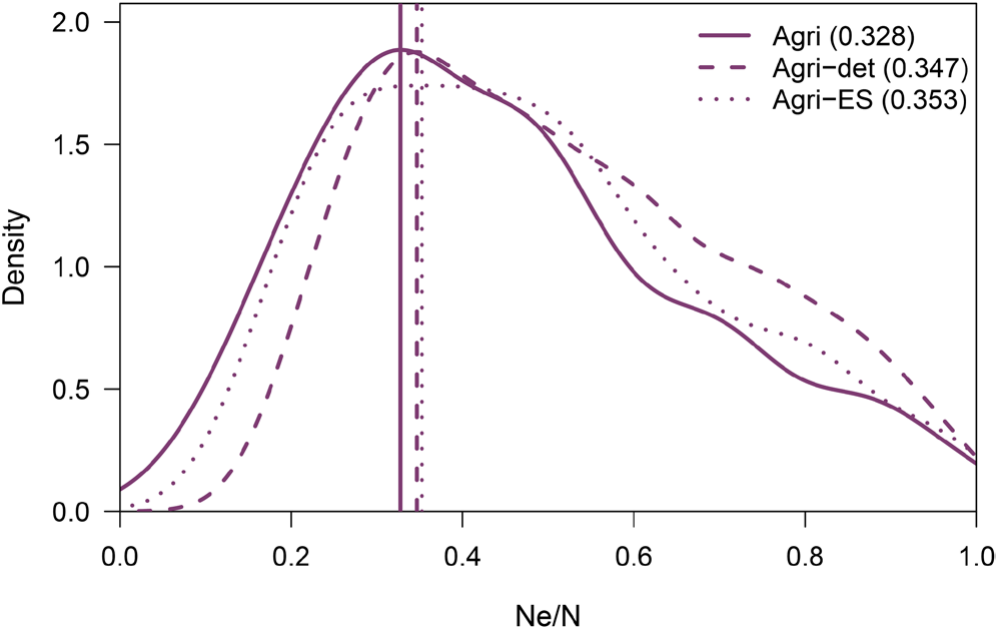
The distribution of *N*_e_/*N* values for 0 < *N*_e_/*N* ≤ 1 in the agricultural baseline model (Agri), the agricultural model without environmental stochasticity (Agri‐det), and the agricultural model with adult environmental stochasticity switched with the forest baseline model. Vertical lines indicate the modal values (density peaks).

When estimating *N*_e_/*N* for each iteration, the results showed three distinct outcomes: population extinction (*N*_e_/*N* = 0), very low genetic drift leading to inflated or negative *N*_e_ estimates (*N*_e_/*N* > 1), and values within the biologically realistic range of 0 < *N*_e_/*N* ≤ 1 (Table 4, Figures 3 and 4). Including environmental stochasticity in the models primarily affected the proportion of iterations resulting in extinction or very low genetic drift, rather than the distribution of *N*_e_/*N* values within the 0–1 interval. The modes of these distributions were similar within each landscape model, both with and without environmental stochasticity.

**TABLE 4:** The proportion of iterations going to extinction (*N*_e_/*N* = 0) and with low genetic drift (*N*_e_/*N* > 1) for the baseline forest (Forest) and agricultural (Agri) models, the baseline models without environmental stochasticity (For‐det, Agri‐det) and the baseline models with environmental stochasticity in adult mortality switched between models (For‐ES, Agri‐ES).

Model	*N*_e_/*N* = 0	*N*_e_/*N* > 1
Forest	12.4%	25.5%
For‐det	0.0%	44.5%
For‐ES	52.6%	10.2%
Agri	69.7%	7.9%
Agri‐det	0.0%	45.3%
Agri‐ES	32.6%	18.1%

In the absence of environmental stochasticity, no iterations resulted in extinction in any of the model runs, but a substantial portion (~45%) experienced very low genetic drift (Table 4). When environmental stochasticity was included, extinctions occurred for both landscape models, but to different extents. In the agricultural baseline model, approximately 70% of iterations resulted in extinction, compared to about 12% in the forest baseline model. Conversely, low‐drift iterations were more common in the forest baseline model (~26%) than in the agricultural baseline model (~8%). Exchanging environmental stochasticity between the models altered these proportions. However, even with reduced environmental stochasticity, the agricultural model still showed more frequent extinctions and fewer low‐drift iterations than the forest baseline model.

### 3.3 Metapopulation Simulations

**FIGURE 5:**
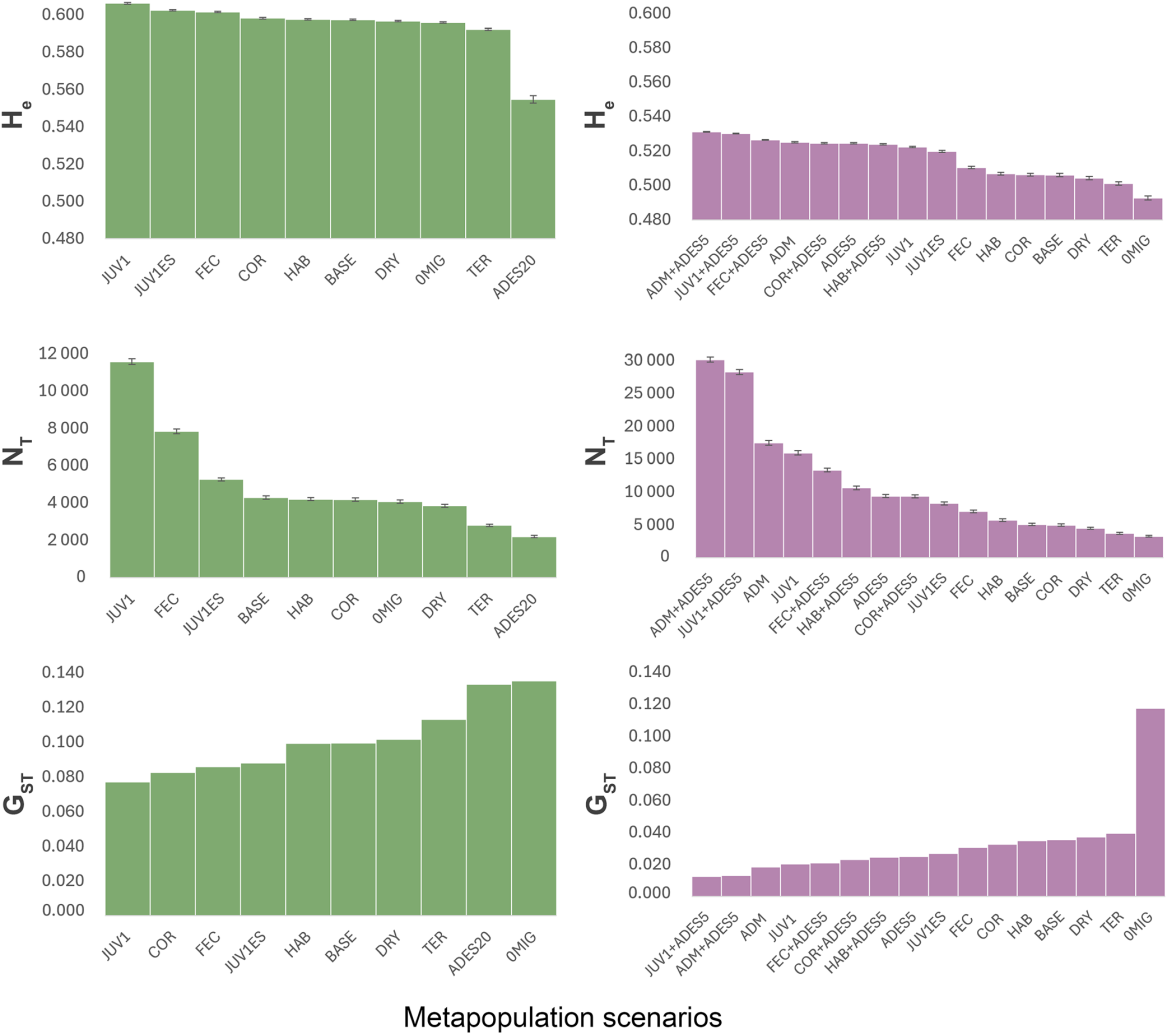
Model outcomes genetic diversity (*H*_e_), total population size (*N*_T_) and genetic differentiation (G_ST_) for the forest (green, left) and agricultural (purple, right) metapopulation scenarios. Abbreviations and scenario descriptions are in Table 2. Error bars represent 95% confidence intervals.

Among the simulated metapopulation scenarios, the highest ranked for the forest metapopulation model was the scenario in which juvenile mortality (age 1) was reduced, and this was consistent for all model outcomes (genetic diversity, total population size, and genetic differentiation) (Figure 5). For the agricultural metapopulation model, scenarios combining reduced environmental stochasticity with either reduced adult mortality or reduced juvenile mortality (age 1) were consistently ranked among the top two highest ranked scenarios across all model outcomes. In the forest metapopulation model, increased environmental stochasticity in adult mortality was the worst‐ranked scenario across all outcomes except genetic differentiation, for which zero migration performed marginally worse. For the agricultural metapopulation model, zero migration was consistently the worst scenario across all model outcomes.

The effect of zero migration was generally more pronounced in the agricultural metapopulation than in the forest metapopulation (Figure 5). For instance, *N*_T_ was reduced by 55% with zero migration in the agricultural metapopulation, but only 5% in the forest metapopulation (Tables S6 and S7). The difference for G_ST_ was even more striking, with a more than 200% increase in the agricultural metapopulation compared to a 36% increase in the forest metapopulation (Figure 5).

At the metapopulation level, the effect of corridor enhancements was noticeable only in the forest model and when genetic differentiation was the model outcome. Habitat enhancements had generally minimal effects in both landscape models and for all model outcomes (Figure 5, Tables S6 and S7). At the subpopulation level, however, the effects of corridor enhancements were pronounced, in particular for genetic diversity (Tables S8 and S10). All 10 forest subpopulations showed an increase in *H*_e_ with corridor enhancements. Only four agricultural subpopulations showed a positive response. However, when corridor enhancements were combined with reduced environmental stochasticity in adult mortality, *H*_e_ increased in six agricultural subpopulations compared to the scenario in which only environmental stochasticity was reduced (Tables S8 and S10). Results for census population size (*N*_T_) at the subpopulation level are provided in Tables S9 and S11.

## 4 Discussion

In this study, using landscape‐specific population models, we show that variation in environmental stochasticity in adult mortality can generate contrasting genetic dynamics. Under high environmental stochasticity, effective population size declined, extinction risk increased, and populations became more dependent on gene flow, while management interventions were less effective. These findings indicate that exposure to environmental stochasticity should be explicitly considered when designing strategies to conserve genetic diversity in amphibian populations and to support long‐term population persistence.

### 4.1 Environmental Stochasticity as a Key Driver of Genetic Erosion

A key difference between the forest and the agricultural model was how the effective population size (*N*_e_) responded to reductions in juvenile mortality or increases in fecundity. The forest model showed a strong increase in *N*_e_, whereas the agricultural model showed only a weak response. In contrast, the models responded more similarly with respect to census population size (*N*_T_). These results likely reflect the higher level of environmental stochasticity in adult mortality in the agricultural model, consistent with previous work showing that environmental stochasticity increases temporal fluctuations in population size (Myhre et al. 2017), thereby increasing genetic drift and reducing *N*_e_ (Allendorf et al. 2022). In our study, both the sensitivity analyses and the metapopulation simulations highlight the importance of environmental stochasticity, particularly for genetic outcomes, suggesting that it is an important driver of the differences between the two landscape models.

When we compared the distribution of *N*_e_/*N* for the baseline models with and without environmental stochasticity, we found that the agricultural model had higher modal *N*_e_/*N*, suggesting that its combination of vital rates was more favorable. This could be due to its more moderate adult mortality, which may have constrained the accumulation of lifetime reproductive success and thereby reduced the variance in reproductive output, which is a key determinant of *N*_e_/*N* in iteroparous species (Waples 2016). Density‐dependent reproduction in the agricultural model may also have elevated *N*_e_/*N* at low densities by increasing the mean reproductive contribution per potential parent, thereby reducing the variance‐to‐mean ratio in reproductive success (Myhre et al. 2016). However, despite the higher modal *N*_e_/*N*, environmental stochasticity strongly reduced average *N*_e_ in the agricultural model. This was likely due to a combination of its effects on the proportion of iterations going extinct (*N*_e_/*N* = 0) and iterations with minimal genetic drift (*N*_e_/*N* > 1), in addition to changes in *N*.

Taken together, environmental stochasticity at the level applied in the agricultural model appears to overwhelm the otherwise positive effects of increased juvenile survival and fecundity, and a more favorable combination of vital rates. Given that this level of stochasticity was derived from real‐life newt populations, this finding is concerning.

A key observation was also that the agricultural metapopulation was more sensitive to population isolation than the forest metapopulation, indicating that stronger genetic drift in the agricultural model enhances the demand for continuous gene flow to maintain genetic diversity. In contrast, the forest metapopulation lost more genetic diversity in the scenario where environmental stochasticity was increased than under population isolation. These findings suggest that threats to genetic diversity may vary with landscape context and depend on levels of environmental stochasticity. However, genetic management of amphibians tends to focus more on the loss of gene flow and connectivity than on reduced resilience to environmental stochasticity (IUCN 2024).

Furthermore, we found that the effects of corridor enhancements, that is, increased connectivity, varied with landscape context. Genetic diversity increased in all forest subpopulations when connectivity was improved, but only in four agricultural subpopulations. However, reducing environmental stochasticity in adult mortality in the agricultural model combined with corridor enhancements led to genetic diversity improving in six agricultural subpopulations, compared to the scenario where only environmental stochasticity was reduced. These findings are consistent with previous studies showing that the effectiveness of genetic management is shaped by the level of population fluctuations (Vucetich and Waite 1999, 2000), which are expected to increase under higher environmental stochasticity (Myhre et al. 2017).

Together, these results suggest that the forest and agricultural models reflect two distinct genetic dynamics. We conceptualize these as “bucket” and “sieve” dynamics, reflecting the system's ability to retain and accumulate genetic diversity. The forest model represents a “bucket” dynamic with high retention of genetic diversity, with relatively low dependence on gene flow but high responsiveness to management efforts. In contrast, the agricultural model behaves more like a “sieve,” where strong genetic drift leads to rapid loss of genetic diversity unless continuously replenished by gene flow, and where responsiveness to management actions is low.

These two distinct dynamics may help explain a previously puzzling empirical result from the same study area. Haugen et al. (2023) found that great crested newt populations in the agricultural landscape had significantly lower genetic diversity than forest populations, despite higher abundance and gene flow. The pattern was tentatively attributed to anthropogenic disturbance and historical founding events. Our results here suggest another plausible explanation: higher exposure to environmental stochasticity in the agricultural populations may have amplified genetic drift, thereby reducing the effectiveness of gene flow in maintaining genetic diversity despite high abundances.

### 4.2 Environmental Stochasticity and Amphibians

Our results indicate that environmental stochasticity can substantially increase genetic drift, thereby reducing effective population sizes. Importantly, the levels of stochasticity applied in our models are not unrealistic for amphibians. High temporal variability in adult survival is frequently reported in amphibian populations (e.g., Brooks et al. 2024; Muths et al. 2018; Pilliod et al. 2022), and in our review of great crested newts (Table S1), 23% of reported estimates showed high temporal variability in adult survival (SD ≥ 20%). This suggests that elevated environmental stochasticity may represent an important threat to conservation of amphibian genetic diversity.

Weather conditions are an important source of environmental stochasticity in amphibians. For example, intense rainfall during hibernation may restrict respiration (Cayuela et al. 2020), mild winters may lead to energy depletion (Griffiths et al. 2010), and cold spells can cause lethal freezing (Muths et al. 2017), leading to year‐to‐year fluctuations in adult survival. Similarly, amphibians' vulnerability to desiccation makes them susceptible to increased mortality during hot, dry summers (Weinbach et al. 2018) and may also reduce food availability (Jaeger 1979).

Although weather conditions are largely beyond human control, their effects on amphibian populations appear to vary depending on local conditions (Cayuela et al. 2016; Muths et al. 2017). Habitats that buffer against adverse weather conditions may therefore be essential for increasing resilience against environmental stochasticity in amphibians.

Given ongoing climate change, with projections of warmer summers, milder winters and more frequent extreme weather events (Bednar‐Friedl et al. 2022), the variability in amphibian vital rates is likely to increase. Our results suggest that this could potentially further reduce effective population sizes in amphibians, primarily through effects on terrestrial life stages, and in particular adult mortality. Although not found to be a key driver in our system, pond drying may also play a role in more drought‐prone systems (Griffiths and Williams 2000; Wang et al. 2011).

### 4.3 Caveats

All models are simplifications of reality but can be useful tools for comparing alternative scenarios and identifying key demographic drivers. Sensitivity analysis 1 showed that both landscape models were sensitive to vital rates, especially fecundity and adult mortality. This sensitivity affects absolute numerical outcomes, but our main focus was on comparing models rather than exact predictions. Importantly, both landscape models highlight the role of environmental stochasticity, and the observations are consistent with established population genetic principles.

In our models, we assumed that juvenile mortality was equal across landscapes. However, juvenile mortality likely also depends on terrestrial habitat quality, similar to adults (Jarvis 2016). While we assumed that adult mortality was affected by habitat loss and degradation, we did not apply similar effects on juvenile mortality. It is therefore plausible that the more continuous forest habitats also support higher juvenile survival, which could lead to larger and more stable populations. Including habitat‐dependent juvenile mortality would likely have strengthened the contrast between landscape models by further reducing genetic drift in the forest model.

Finally, when estimating mean model outcomes across all iterations, we chose to include both extinct and extant populations. Excluding iterations that ended in extinction would have disproportionately removed poor‐performing iterations in the agricultural model, thus introducing survival bias. This means that the mean model outcomes reflect both changes in genetic diversity and variation in extinction rates.

### 4.4 Management Implications

The genetic dynamics observed in our study have important implications for management. “Bucket” populations, represented here by forest populations, may appear genetically robust under current conditions, but are inherently vulnerable to increased exposure to environmental stochasticity. Unlike agricultural populations, the forest populations may not be able to persist in a “sieve” state where high genetic drift is compensated for by continuous gene flow. Several factors contribute to this constraint: marginal breeding habitats often have low potential for compensatory recruitment (Cayuela et al. 2022; Muths et al. 2011), and reduced reproduction entails fewer potential migrants, resulting in lower dispersal rates and higher susceptibility to landscape barriers (Haugen et al. 2023).

The agricultural populations characterized by “sieve” dynamics, that is, high genetic drift and stronger reliance on gene flow to maintain genetic diversity, present other management challenges. Although current dispersal rates are relatively high in the agricultural landscape due to low‐resistance land use (e.g., grass production) (Haugen et al. 2023), future land‐use changes could reduce connectivity and increase vulnerability to genetic erosion. In addition, as long as the populations display “sieve” dynamics, the genetic effects of management efforts, such as translocation (Gustafsson et al. 2016) or increased connectivity (O'Brien et al. 2021), may be uncertain and less predictable.

Our study emphasizes the importance of maintaining and reducing exposure to environmental stochasticity to preserve genetic diversity. This may be achieved by preserving and restoring microhabitats that enhance survival during periods of harsh weather. Importantly, habitat split, that is, when resources required to complete the life cycle are separated by hostile matrix (Becker et al. 2010), poses an additional threat by reducing habitat availability and forcing newts out of buffering microhabitats, thereby increasing their exposure to weather conditions. Therefore, habitat split should be addressed, and creating corridors between vital resources could be a viable strategy to mitigate this problem.

However, the distribution of suitable habitat may shift under climate change, which may challenge static conservation strategies (Chiocchio et al. 2024). While access to buffering microhabitats may reduce exposure to environmental stochasticity in the short term, long‐term persistence may also depend on the ability of populations to track shifting climatic conditions (Souza et al. 2023; Tiberti et al. 2021). This suggests that management strategies should not only maintain access to buffering microhabitats but also allow for movement across the landscape.

In summary, our results show that environmental stochasticity can lead to distinct genetic dynamics that entail different management priorities. Under high stochasticity, connectivity alone may be insufficient to maintain genetic diversity, and efforts to increase resilience to environmental variability may be equally important. Recognizing whether populations function as “buckets” or “sieves” may help guide landscape‐specific conservation strategies.

## Funding

The authors have nothing to report.

## Conflicts of Interest

The authors declare no conflicts of interest.

## Supporting information


**Figure S1:** Results from sensitivity analysis 2. Changes in effective population size (*N*_e_) with incremental changes in input parameters in the forest (green) and agricultural (purple) model.
**Figure S2:** Results from sensitivity analysis 2. Changes in total population size (*N*_T_) with incremental changes in input parameters in the forest (green) and agricultural (purple) model.
**Figure S3:** Results from sensitivity analysis 2. Changes in heterozygosity (H_e_) with incremental changes in input parameters in the forest (green) and agricultural (purple) model.
**Table S1:** Survival estimates for adult and juvenile survival in *Triturus cristatus* collected from literature.
**Table S2:** Data from the metapopulation simulations. Initial population size (juveniles and adults) calculated from catch per unit effort data from the same year as genetic sampling, and habitat carrying capacity (K) for the baseline and the habitat enhancement scenario (HAB).
**Table S3:** Dispersal distances applied in the forest metapopulation model (left) and the agricultural metapopulation model (right). Pop = population ID, disp.base = dispersal rates used in the baseline scenarios, disp.COR = dispersal rates in the corridor enhancement scenarios. In addition, geographical distance (meters) between each population (geo.dist).
**Table S4:** Results from sensitivity analysis 1 where input parameters are changed by ±5. Input parameters ranked after effect size of parameter change using linear regression slope.
**Table S5:** Results from sensitivity analysis 2. Values represent difference in outcomes from baseline model and the models with maximum (gray) and minimum (white) parameter input according to realistic ranges.
**Table S6:** Simulation results from the forest metapopulation scenarios. Green showing baseline scenario.
**Table S7:** Simulation results from the agricultural metapopulation scenarios. Purple showing baseline scenario.
**Table S8:** Simulation results for heterozygosity in the forest subpopulations. Green showing baseline scenario.
**Table S9:** Simulation results for *N*_T_ for the forest subpopulations. Green showing baseline scenario.
**Table S10:** Simulation results for *H*_e_ for the agricultural subpopulations. Purple showing baseline scenario.
**Table S11:** Simulation results for *N*_T_ for the agricultural subpopulations. Purple showing baseline scenario.

## Data Availability

Data sharing not applicable to this article as no datasets were generated or analyzed during the current study.
