# Supplementary material for: Landscape Context and Environmental Stochasticity Shape Genetic Management Outcomes in an Amphibian
Source: Evol Appl. 2026 Aug 5;19(8):e70310. doi: 10.1111/eva.70310 (PMC13443712; doi:10.1111/eva.70310)
Supplement: Supplementary file 1 [file EVA-19-e70310-s001.docx]

**Supporting information**

**Landscape context and environmental stochasticity shape genetic management outcomes in an amphibian.**

Hanne Haugen, Børre K. Dervo, Arne Linløkken, Jan Heggenes and Kjartan Østbye

**Detailed description of density dependent effects in the PVA-models**

*Density-dependent fecundity*

We created a formula for density-dependent fecundity using data on *Triturus cristatus* and *Ichthyosaura alpestris* (where data on *T. cristatus* was lacking):

$$100\times\left( -0.0006\times\left( \frac{B\times100}{A} \right)+0.1095 \right)$$

where B is the number of broods and A is pond area (m^2^).

Here, we describe how we arrived at the above formula:

1. Larval survival in *I. alpestris* ranges from 0.94 to 0.78 when going from low to high larval density conditions (Van Buskirk, 2007).
2. Fecundity is the result of both egg and larval survival. *T.cristatus* has a daily egg survival of 0.88 (Miaud, 1994). At 17 ° C, egg hatching typically takes 15 days (Griffithsand & Wijer, 1994). This means that total egg survival is 0.147.
3. We multiplied the density-conditional larval survival from *I.alpetris* by the egg survival rate from *T.cristatus* to estimate the total egg/larval survival rate under low and high density conditions (0.140 and 0.115, respectively).
4. Low density and high density were defined as 10.8 and 35.5 *I.alpetris* larvae per m^3^. Based on body mass, 6 *T. alpestris* larval are equivalent to 1.5 *T.cristatus* larvae (Van Buskirk, 2007).
5. We then estimated the equivalent egg density by diving larval density by egg survival and found that low egg density represented 15 eggs per m^3^ and high density represented 61 eggs per m^3^ for *T.cristatus.*
6. We plotted the number of eggs against survival rates and drew a linear function between the two points, and found that the slope was -0.0006, representing the reduction in egg/larval survival per egg added to the egg density.
7. The maximum density in agricultural ponds was 100 eggs per m^2^.
8. The only published estimate of egg/larval survival in *T. cristatus* is 5% (Oldham, 1994). We assumed this represented high-density conditions. We inserted 100 eggs into the linear function and calculated the intercept, assuming 5% survival at this density. The intercept was found to be 10.95.
9. Thus, our formula estimated fecundity to be 5 metamorps per female at high density and 11 metamorps at low density.

*The percentage of breeding females determined by aquatic vegetation cover*

The percentage of breeding females were modelled as density dependent and depending on the cover of aquatic vegetation. Using aerial photos and field observations, we delimited the area of aquatic vegetation cover and calculated the maximum number of breeding females (F_max_) per pond. A density dependent function was used to determine annual percentage of breeding females, where up to F_max_, all mature females bred. When the number of mature females (F) exceed F_max_, the percentage of breeding females were calculated as F_max_/F ×100. Due to denser vegetation in the agricultural ponds, we assumed that one female required 1 m^2^ to lay all her eggs, but would need 2 m^2^ in a forest pond where aquatic vegetation was more sparse.

**Table S1:** Survival estimates for adult and juvenile survival in *Triturus cristatus* collected from literature.

| **Adult survival** | **Survival SD** | **Years** | **Landscape context** | **Country** | **Reference** | **Comments** |
| --- | --- | --- | --- | --- | --- | --- |
| 87.0 % | 2.2 % | 7 |  | France | Cayuela et al. (2020) | SD estimated from survival estimates collected from Figure 2 in reference. |
| 83.0 % | 6.4 % | 16 | Gardens, hedgerows, trees, sport field. | UK | Cayuela et al. (2020) | SD estimated from survival estimates collected from Figure 2 in reference. |
| 75.0 % |  | 4 | Small farm, surrounded by forest | Norway | Nergaard (2020) |  |
| 71.0 % | 5.0 % | 4 | Park, flower beds, forest. | Sweden | Hagström (1979) |  |
| 70.0 % | 11.0 % | 6 | Small farm, surrounded by forest. | Norway | Rudshaug (2024) |  |
| 69.0 % | 24.0 % | 7 | Park, lawns. | UK | Baker (1999) |  |
| 65.0% |  | 10 | Roads, houses, arable fields. | Norway | Dervo. B., unpublished work |  |
| 62.0 % | 23.0 % | 15 |  | France | Cayuela et al. (2017) | SD estimated from survival estimates collected from Figure 5 in reference. |
| 62.0 % |  | 7 |  | France | Cogălniceanu, Stănescu, and Arntzen (2020) |  |
| 62.0 % | 20.0 % | 15 | Mediterranean area, open area and forest. | France | Cayuela et al. (2020) | Minimum and maximum survival estimates collected from Figure 2. SD estimated as (max survival - min survival)/4 |
| 62.0 % | 16.0 % | 18 | Agricultural landscape. | UK | Cayuela et al. (2020) | Minimum and maximum survival estimates collected from Figure 2. SD estimated as (max survival - min survival)/4 |
| 55.0 % | 14.0% | 11 | Mixed arable land and orchards. hedgerows. | UK | Griffiths, Sewell, and McCrea (2010) | SD estimated as (max survival - min survival)/4 |
| 49.0 % |  | 4 |  | UK | Jarvis (2012) |  |
| 48.0 % | 6.0 % | 6 | Abandoned sand quarry, coastal dune area. | France | Arntzen and Teunis (1993) | SD estimated as (max survival - min survival)/4 |
| 29.0 % | 12.0 % | 19 | Forest, nature reserve. | France | Cayuela et al. (2020) | Minimum and maximum survival estimates collected from Figure 2. SD estimated as (max survival - min survival)/4 |
| 29.0 % | 11.0 % | 19 |  | France | Cayuela et al. (2017) | Minimum and maximum survival estimates collected from Figure 5. SD estimated as (max survival - min survival)/4 |
| 26.0 % | 16.0 % | 18 | Agricultural landscape | Germany | Bülow and Kupfer (2019) | SD estimated as (max survival - min survival)/4 |
|  |  |  |  |  |  |  |
| **Juvenile survival** | **Survival SD** | **Years** | **Landscape context** | **Country** | **Reference** | **Comments** |
| 84.0 % |  | 3 |  | UK | Cummings and Swan (2000) |  |
| 59.0 % |  | 7 |  | UK | Baker (1999) |  |
| 46.0 % | 24.0 % | 15 |  | France | Cayuela et al. (2017) | SD estimated from survival estimates collected from Figure 5. |
| 32.0 % | 23.0 % | 15 |  | France | Cayuela et al. (2017) | SD estimated from survival estimates collected from Figure 5. |
| 32.0 % | 11.0 % | 19 |  | France | Cayuela et al. (2017) | SD estimated from survival estimates collected from Figure 5. |
| 24.0 % | 17.0 % | 19 |  | France | Cayuela et al. (2017) | SD estimated from survival estimates collected from Figure 5. |
| 22.0 % | 10.0 % | 6 | Abandoned sand quarry, coastal dune area. | France | Arntzen and Teunis (1993) | SD estimated as (max survival - min survival)/4. |
| 19.6 % |  |  | Forests, pastures. | UK | Jarvis (2012) |  |

**Table S2:** Data from the metapopulation simulations. Initial population size (juveniles and adults) calculated from catch per unit effort data from the same year as genetic sampling, and habitat carrying capacity (K) for the baseline and the habitat enhancement scenario (HAB).

| **Pop ID** | **Landscape context** | **Int. pop. size** | **K baseline** | **K scenario HAB** |
| --- | --- | --- | --- | --- |
| A | Forest | 1 222 | 15 120 | 19 050 |
| B | Forest | 4 378 | 19 520 | 14 900 |
| C | Forest | 668 | 22 188 | 21 258 |
| D | Forest | 460 | 38 022 | 40 828 |
| E | Forest | 1 080 | 16 724 | 14 974 |
| G | Forest | 168 | 18 760 | 19 110 |
| H | Forest | 2 134 | 20 156 | 24 876 |
| I | Forest | 1 088 | 12 472 | 12 936 |
| J | Forest | 1 068 | 9 420 | 9 380 |
| M | Forest | 914 | 8 094 | 12 210 |
| 76 | Agricultural | 5 874 | 15 590 | 19 860 |
| 94 | Agricultural | 1 614 | 10 116 | 17 298 |
| 95 | Agricultural | 8 452 | 10 464 | 16 746 |
| 127 | Agricultural | 5 392 | 5 402 | 13 524 |
| 128 | Agricultural | 508 | 5 402 | 13 486 |
| 138 | Agricultural | 596 | 46 430 | 48 910 |
| 139 | Agricultural | 1 900 | 24 330 | 30 926 |
| 487 | Agricultural | 966 | 28 072 | 36 006 |
| 488 | Agricultural | 3 532 | 8 714 | 16 904 |
| 494 | Agricultural | 138 | 32 222 | 37 954 |

**Calculation of effective population size (N_e_)**

Effective population size was calculated based on equation 1 from Holleley et al. (2014), where H0 and Ht are heterozygosity at year 0 and year 50, respectively, and t is the number of generations :

$$\Delta He=\frac{Ht}{H0}=\left( 1-\frac{1}{2Ne} \right)^{t}$$

Solving for N_e_ gives

$$Ne= \frac{1}{2\left( 1-\left( \frac{Ht}{H0} \right)^{\frac{1}{t}} \right)}$$

**Table S3**: Dispersal distances applied in the forest metapopulation model (left) and the agricultural metapopulation model (right). Pop = population ID, disp.base = dispersal rates used in the baseline scenarios, disp.COR = dispersal rates in the corridor enhancement scenarios. In addition, geographical distance (meters) between each population (geo.dist).

| *Forest* | | | | |  | *Agricultural* | | | | |
| --- | --- | --- | --- | --- | --- | --- | --- | --- | --- | --- |
| **Pop** | **Pop** | **disp.base** | **disp.COR** | **geo. dist.** |  | **Pop** | **Pop** | **disp.base** | **disp.COR** | **geo. dist.** |
| a | b | 0.22 % | 0.46 % | 1 153 |  | 76 | 94 | 0.00 % | 0.00 % | 4 484 |
| a | c | 0.19 % | 0.42 % | 1 168 |  | 76 | 95 | 0.49 % | 0.66 % | 3 044 |
| a | d | 0.22 % | 0.47 % | 2 045 |  | 76 | 127 | 0.77 % | 0.86 % | 3 033 |
| a | e | 0.00 % | 0.00 % | 3 193 |  | 76 | 128 | 0.00 % | 0.00 % | 3 254 |
| a | g | 0.15 % | 0.52 % | 1 256 |  | 76 | 138 | 2.90 % | 2.96 % | 1 319 |
| a | h | 0.20 % | 0.41 % | 677 |  | 76 | 139 | 0.74 % | 1.00 % | 2 683 |
| a | i | 0.00 % | 0.00 % | 5 641 |  | 76 | 487 | 0.39 % | 0.41 % | 1 994 |
| a | j | 0.00 % | 0.00 % | 5 818 |  | 76 | 488 | 0.54 % | 0.66 % | 1 658 |
| a | m | 0.00 % | 0.00 % | 4 115 |  | 76 | 494 | 0.34 % | 0.88 % | 2 765 |
| b | c | 0.32 % | 0.52 % | 1 545 |  | 94 | 95 | 0.12 % | 0.21 % | 2 573 |
| b | d | 0.00 % | 0.00 % | 3 190 |  | 94 | 127 | 0.00 % | 0.00 % | 7 431 |
| b | e | 0.00 % | 0.00 % | 4 286 |  | 94 | 128 | 0.00 % | 0.00 % | 7 583 |
| b | g | 0.17 % | 0.48 % | 2 265 |  | 94 | 138 | 0.00 % | 0.00 % | 3 711 |
| b | h | 0.22 % | 0.37 % | 1 647 |  | 94 | 139 | 0.00 % | 0.00 % | 4 417 |
| b | i | 0.00 % | 0.00 % | 6 634 |  | 94 | 487 | 0.00 % | 0.00 % | 5 275 |
| b | j | 0.00 % | 0.00 % | 6 890 |  | 94 | 488 | 0.00 % | 0.00 % | 5 606 |
| b | m | 0.00 % | 0.00 % | 5 092 |  | 94 | 494 | 0.00 % | 0.00 % | 6 750 |
| c | d | 0.24 % | 0.44 % | 2 316 |  | 95 | 127 | 0.00 % | 0.00 % | 5 479 |
| c | e | 0.00 % | 0.00 % | 3 883 |  | 95 | 128 | 0.00 % | 0.00 % | 5 540 |
| c | g | 0.15 % | 0.44 % | 2 250 |  | 95 | 138 | 0.85 % | 1.16 % | 1 787 |
| c | h | 0.20 % | 0.37 % | 815 |  | 95 | 139 | 0.40 % | 0.67 % | 1 874 |
| c | i | 0.00 % | 0.00 % | 6 452 |  | 95 | 487 | 0.18 % | 0.22 % | 2 966 |
| c | j | 0.00 % | 0.00 % | 6 481 |  | 95 | 488 | 0.00 % | 0.00 % | 3 508 |
| c | m | 0.00 % | 0.00 % | 4 980 |  | 95 | 494 | 0.00 % | 0.00 % | 4 529 |
| d | e | 0.32 % | 0.57 % | 1 761 |  | 127 | 128 | 0.58 % | 1.79 % | 377 |
| d | g | 0.19 % | 0.65 % | 1 537 |  | 127 | 138 | 0.00 % | 0.00 % | 3 790 |
| d | h | 0.24 % | 0.43 % | 1 637 |  | 127 | 139 | 0.00 % | 0.00 % | 4 092 |
| d | i | 0.00 % | 0.00 % | 4 343 |  | 127 | 487 | 0.20 % | 0.24 % | 2 677 |
| d | j | 0.00 % | 0.00 % | 4 229 |  | 127 | 488 | 0.31 % | 0.40 % | 1 982 |
| d | m | 0.09 % | 0.22 % | 3 027 |  | 127 | 494 | 0.26 % | 0.74 % | 1 349 |
| e | g | 0.16 % | 0.48 % | 2 040 |  | 128 | 138 | 0.00 % | 0.00 % | 3 903 |
| e | h | 0.18 % | 0.33 % | 3 090 |  | 128 | 139 | 0.00 % | 0.00 % | 4 058 |
| e | i | 0.10 % | 0.26 % | 2 597 |  | 128 | 487 | 0.09 % | 0.16 % | 2 657 |
| e | j | 0.10 % | 0.21 % | 2 628 |  | 128 | 488 | 0.12 % | 0.22 % | 2 035 |
| e | m | 0.10 % | 0.23 % | 1 298 |  | 128 | 494 | 0.13 % | 0.49 % | 1 189 |
| g | h | 0.15 % | 0.43 % | 1 462 |  | 138 | 139 | 1.48 % | 2.12 % | 1 575 |
| g | i | 0.00 % | 0.00 % | 4 396 |  | 138 | 487 | 0.64 % | 0.66 % | 1 646 |
| g | j | 0.00 % | 0.00 % | 4 626 |  | 138 | 488 | 0.48 % | 0.62 % | 1 897 |
| g | m | 0.07 % | 0.21 % | 2 863 |  | 138 | 494 | 0.41 % | 1.07 % | 3 042 |
| h | i | 0.00 % | 0.00 % | 5 645 |  | 139 | 487 | 0.42 % | 0.53 % | 1 415 |
| h | j | 0.00 % | 0.00 % | 5 704 |  | 139 | 488 | 0.24 % | 0.38 % | 2 176 |
| h | m | 0.00 % | 0.00 % | 4 166 |  | 139 | 494 | 0.28 % | 0.82 % | 2 921 |
| i | j | 0.08 % | 0.22 % | 999 |  | 487 | 488 | 0.17 % | 0.24 % | 803 |
| i | m | 0.07 % | 0.19 % | 1 543 |  | 487 | 494 | 0.20 % | 0.43 % | 1 569 |
| j | m | 0.06 % | 0.15 % | 2 012 |  | 488 | 494 | 0.19 % | 0.54 % | 1 177 |
| **Median** | | **0.07 %** | **0.21 %** | **2 863** |  | **Median** | | **0.18 %** | **0.24 %** | **2 765** |
| **SD** |  | **0.10 %** | **0.21 %** | **1 822** |  | **SD** |  | **0.50 %** | **0.61 %** | **1 733** |

**Description of landscape modulated dispersal rates**

We used remote sensing data (LiDAR) and natural resource maps to model current and future landscape properties relevant for dispersal rates and carrying capacity. In the forest landscape, we modelled vegetation types based on their drought tolerance to represent moisture conditions. Moisture conditions were modeled using the Gradient Boosted tool in ArcGIS Pro and predictors from Haugen, Devineau, Heggenes, Østbye, and Linløkken (2022), including sediment types, topographic position, soil depth, and the Light Penetration Index (LPI). LPI is a LiDAR-based index that represents the amount of light reaching the ground beneath the forest canopy (Bode, Limm, Power, & Finlay, 2014). We also modeled the amount of field vegetation cover using a beta regression model from Haugen et al. (2022), including forest type and LPI as predictors. In the agricultural landscape, we focused on land cover types using existing land cover maps, which we manually updated to reflect conditions during genetic sampling in 2021.

In the management scenarios representing corridor enhancements, we aimed to model the effect of improved landscape permeability between populations. In the forest landscape, we modeled moisture conditions under a continuous canopy cover. We used the LPI index and modeled how LPI would vary in areas with clear-cuts and young forests, assuming these areas instead were mature forests. We used predictors related to topography, and forest and soil properties to model LPI in these areas, including Saga Wetness index (Boehner & Selige, 2006), aspect, slope, standardized topographic position index (Gallant & Wilson, 2000), soil depth, forest type and soil productivity. The Forest-based classification tool in ArcGIS pro was used to create this new LPI map. We then applied the same procedure as described earlier to generate a moisture map, but now using the altered LPI index. In the agricultural landscape, we simply added 30 m wide corridors to the current landscape cost surface.

To estimate dispersal rates, we used the moisture map from the forest landscape and included land cover (primarily gravel roads). In the agricultural landscape, we used land cover maps. We then assigned cost values based on moisture levels and land cover types, in accordance with a previous study conducted in the same study areas (Haugen et al., 2023)

The cost surfaces were used to estimate movement costs between each population pair using ResistanceGA (Peterman, 2018), which implements Circuitscape (Anantharaman, Hall, Shah, & Edelman, 2020). These movement costs were then used to weight dispersal rates between populations according to the following formula:

$$D \times\left( \frac{{Cost}_{ij}}{{Cost}_{min}} \right)^{-r}$$

where D is a baseline dispersal rate prior to adjusting for movement cost, Cost_min_ is the lowest movement cost found within the current landscape, Cost_ij_ is the movement cost between pond i and j, and r is the rate of change.

Since baseline dispersal rate (D) and rate of change (r) were unknown, we adjusted the formula to closely match the mean, median, minimum and maximum migration rates calculated in the previous study from genetic data (Haugen et al., 2023). This resulted in D and r being 0.0032 and −1.1 in the forested area and 0.029 and −3.9 in the agricultural area, respectively. Dispersal was assumed to be 0 when distance between ponds exceeded ~3 km.

Habitat capacity was estimated by delineating areas of suitable habitat, defined as semi-moist to moist vegetation with at least 30 % field vegetation cover in the forested landscape, and forest and uncultivated fields in the agricultural landscape. The initial population sizes were divided by the total habitat area for each population to calculate the maximum observed density. This maximum density was then multiplied by the habitat area of each population to estimate the terrestrial habitat carrying capacity.

In the habitat enhancement scenario for the forest populations, we used modeled moisture and field vegetation cover maps based on the altered LPI index to delineate areas of suitable habitat under a continuous canopy forest scenario. For the agricultural populations, we added a 100 m radius of suitable habitat around the breeding ponds.

Resistance surface maps from the forest landscape for the baseline scenario (left) and the corridor enhancement scenarios (right). Yellow areas indicate high movement cost, green indicate intermediate cost and blue indicates low cost. Pink dots mark the locations of great crested newt populations.

Resistance surface maps from the agricultural landscape for the baseline scenario (left) and the corridor enhancement scenarios (right). Black lines represent 30 m wide corridors. Yellow areas indicate high movement cost, green indicate intermediate cost and blue indicates low cost. Pink dots mark the locations of great crested newt populations.

**Table S4:** Results from sensitivity analysis 1 where input parameters are changed by ± 5. Input parameters ranked after effect size of parameter change using linear regression slope.

| **Rank** | **Parameters** | **Forest** | **Parameters** | **Agricultural** |
| --- | --- | --- | --- | --- |
|  |  | *H_e_* |  | *H_e_* |
| 1 | Fecundity | 0.260 | Fecundity | 0.111 |
| 2 | Adult mortality | 0.109 | Adult mortality | 0.081 |
| 3 | Juvenile mortality (age 1) | 0.056 | ES in adult mortality | 0.067 |
| 4 | Juvenile mortality (age 2) | 0.042 | Juvenile mortality (age 1) | 0.049 |
| 5 | ES in adult mortality | 0.034 | Juvenile mortality (age 2) | 0.043 |
| 6 | Juvenile mortality (age 4) | 0.026 | Juvenile mortality (age 3) | 0.036 |
| 7 | Juvenile mortality (age 3) | 0.023 | ES in juvenile mortality (age 2) | 0.028 |
| 8 | ES in juvenile mortality (age 1) | 0.019 | ES in juvenile mortality (age 1) | 0.024 |
| 9 | ES in juvenile mortality (age 2) | 0.017 | ES in juvenile mortality (age 3) | 0.020 |
| 10 | ES in juvenile mortality (age 3) | 0.014 | Juvenile mortality (age 4) | 0.016 |
| 11 | ES in juvenile mortality (age 4) | 0.009 | ES in juvenile mortality (age 4) | 0.016 |
| 12 | Carrying capacity | 0.005 | Carrying capacity | 0.006 |
| 13 | Aquatic vegetation | 0.001 | SD in fecundity | 0.002 |
| 14 | SD in fecundity | 0.001 | Aquatic vegetation | 0.000 |
|  |  | *N_e_* |  | *N_e_* |
| 1 | Fecundity | 43.90 | Fecundity | 2.28 |
| 2 | Adult mortality | 20.71 | Adult mortality | 1.50 |
| 3 | Juvenile mortality (age 1) | 8.58 | ES in adult mortality | 1.20 |
| 4 | Juvenile mortality (age 2) | 6.58 | Juvenile mortality (age 1) | 0.94 |
| 5 | ES in adult mortality | 5.17 | Juvenile mortality (age 2) | 0.81 |
| 6 | Juvenile mortality (age 4) | 3.91 | Juvenile mortality (age 3) | 0.72 |
| 7 | Juvenile mortality (age 3) | 3.62 | ES in juvenile mortality (age 2) | 0.50 |
| 8 | ES in juvenile mortality (age 1) | 2.69 | ES in juvenile mortality (age 1) | 0.46 |
| 9 | ES in juvenile mortality (age 2) | 2.59 | ES in juvenile mortality (age 3) | 0.36 |
| 10 | ES in juvenile mortality (age 3) | 2.41 | Juvenile mortality (age 4) | 0.30 |
| 11 | ES in juvenile mortality (age 4) | 1.39 | ES in juvenile mortality (age 4) | 0.29 |
| 12 | Carrying capacity | 0.71 | Carrying capacity | 0.11 |
| 13 | Aquatic vegetation | 0.19 | SD in fecundity | 0.03 |
| 14 | SD in fecundity | 0.07 | Aquatic vegetation | 0.01 |
|  |  | *N_T_* |  | *N_T_* |
| 1 | Fecundity | 1 197.3 | Fecundity | 308.05 |
| 2 | Adult mortality | 360.7 | Adult mortality | 204.36 |
| 3 | Juvenile mortality (age 1) | 237.7 | Juvenile mortality (age 2) | 129.41 |
| 4 | Juvenile mortality (age 2) | 173.8 | Juvenile mortality (age 1) | 125.15 |
| 5 | Juvenile mortality (age 3) | 103.2 | Juvenile mortality (age 3) | 87.23 |
| 6 | Juvenile mortality (age 4) | 76.6 | ES in adult mortality | 58.67 |
| 7 | ES in juvenile mortality (age 2) | 33.2 | Juvenile mortality (age 4) | 26.63 |
| 8 | ES in adult mortality | 16.7 | ES in juvenile mortality (age 1) | 26.08 |
| 9 | Carrying capacity | 10.7 | ES in juvenile mortality (age 4) | 26.05 |
| 10 | Aquatic vegetation | 10.2 | Carrying capacity | 22.61 |
| 11 | ES in juvenile mortality (age 3) | 6.5 | ES in juvenile mortality (age 2) | 17.03 |
| 12 | ES in juvenile mortality (age 4) | 5.9 | Aquatic vegetation | 14.48 |
| 13 | ES in juvenile mortality (age 1) | 3.9 | ES in juvenile mortality (age 3) | 9.62 |
| 14 | SD in fecundity | 1.3 | SD in fecundity | 1.69 |

**Table S5:** Results from sensitivity analysis 2. Values represent difference in outcomes from baseline model and the models with maximum (grey) and minimum (white) parameter input according to realistic ranges.

| **Rank** | **Parameters** | **Forest** | **Parameters** | **Agricultural** |
| --- | --- | --- | --- | --- |
|  |  | *H_e_* |  | *H_e_* |
| 1 | Juvenile mortality (age 1) | 0.109 | Juvenile mortality (age 1) | 0.309 |
| 2 | Fecundity | 0.103 | Adult mortality | 0.258 |
| 3 | Juvenile mortality (age 2) | 0.093 | Fecundity | 0.213 |
| 4 | Juvenile mortality (age 3) | 0.078 | Juvenile mortality (age 2) | 0.210 |
| 5 | ES in juvenile mortality (age 1) | 0.072 | ES in adult mortality | 0.202 |
| 6 | ES in juvenile mortality (age 2) | 0.06 | ES in juvenile mortality (age 1) | 0.184 |
| 7 | Juvenile mortality (age 4) | 0.05 | Juvenile mortality (age 3) | 0.117 |
| 8 | ES in adult mortality | 0.04 | ES in juvenile mortality (age 2) | 0.109 |
| 9 | Adult mortality | 0.04 | ES in juvenile mortality (age 3) | 0.080 |
| 10 | ES in juvenile mortality (age 3) | 0.03 | Juvenile mortality (age 4) | 0.077 |
| 11 | ES in juvenile mortality (age 4) | 0.03 | ES in juvenile mortality (age 4) | 0.035 |
| 12 | SD in fecundity | 0.01 | Aquatic vegetation | 0.025 |
| 13 | Carrying capacity | 0.00 | ES in juvenile mortality (age 1) | 0.008 |
| 14 | Aquatic vegetation | - 0.00 | Carrying capacity | 0.002 |
| 15 | SD in fecundity | - 0.00 | ES in juvenile mortality (age 3) | 0.001 |
| 16 | ES in juvenile mortality (age 2) | - 0.01 | ES in juvenile mortality (age 4) | 0.001 |
| 17 | ES in juvenile mortality (age 1) | - 0.01 | SD in fecundity | - 0.002 |
| 18 | ES in juvenile mortality (age 4) | - 0.02 | SD in fecundity | - 0.014 |
| 19 | ES in juvenile mortality (age 3) | - 0.03 | ES in juvenile mortality (age 2) | - 0.014 |
| 20 | Juvenile mortality (age 1) | - 0.24 | ES in adult mortality | - 0.029 |
| 21 | ES in adult mortality | - 0.27 | Fecundity | - 0.102 |
| 22 | Juvenile mortality (age 2) | - 0.38 | Juvenile mortality (age 1) | - 0.112 |
| 23 | Juvenile mortality (age 3) | - 0.41 | Carrying capacity | - 0.140 |
| 24 | Juvenile mortality (age 4) | - 0.42 | Juvenile mortality (age 4) | - 0.140 |
| 25 | Fecundity | - 0.43 | Aquatic vegetation | - 0.144 |
| 26 | Adult mortality | - 0.44 | Juvenile mortality (age 2) | - 0.148 |
| 27 | Carrying capacity | - 0.44 | Adult mortality | - 0.149 |
| 28 | Aquatic vegetation | - 0.44 | Juvenile mortality (age 3) | - 0.158 |
|  |  | *N_e_* |  | *N_e_* |
| 1 | Fecundity | 288.99 | Juvenile mortality (age 1) | 24.05 |
| 2 | Juvenile mortality (age 1) | 282.47 | Adult mortality | 8.98 |
| 3 | Juvenile mortality (age 2) | 161.66 | Juvenile mortality (age 2) | 8.37 |
| 4 | Juvenile mortality (age 3) | 35.54 | ES in adult mortality | 7.17 |
| 5 | ES in juvenile mortality (age 1) | 34.67 | ES in juvenile mortality (age 1) | 6.09 |
| 6 | ES in juvenile mortality (age 2) | 18.45 | Fecundity | 6.04 |
| 7 | Juvenile mortality (age 4) | 12.86 | Juvenile mortality (age 3) | 3.18 |
| 8 | ES in adult mortality | 8.83 | ES in juvenile mortality (age 2) | 2.71 |
| 9 | ES in juvenile mortality (age 3) | 7.68 | ES in juvenile mortality (age 3) | 1.76 |
| 10 | Adult mortality | 7.38 | Juvenile mortality (age 4) | 1.19 |
| 11 | ES in juvenile mortality (age 4) | 4.58 | ES in juvenile mortality (age 4) | 0.73 |
| 12 | SD in fecundity | 0.79 | Aquatic vegetation | 0.46 |
| 13 | Carrying capacity | 0.48 | ES in juvenile mortality (age 1) | 0.15 |
| 14 | Fecundity | - | Carrying capacity | 0.04 |
| 15 | SD in fecundity | - 0.61 | ES in juvenile mortality (age 3) | 0.02 |
| 16 | Aquatic vegetation | - 0.66 | ES in juvenile mortality (age 4) | 0.01 |
| 17 | ES in juvenile mortality (age 2) | - 1.18 | Fecundity | - |
| 18 | ES in juvenile mortality (age 1) | - 1.36 | SD in fecundity | - 0.03 |
| 19 | ES in juvenile mortality (age 4) | - 1.95 | SD in fecundity | - 0.24 |
| 20 | ES in juvenile mortality (age 3) | - 4.33 | ES in juvenile mortality (age 2) | - 0.25 |
| 21 | Juvenile mortality (age 1) | - 10.25 | ES in adult mortality | - 0.50 |
| 22 | ES in adult mortality | - 10.60 | Juvenile mortality (age 1) | - 1.86 |
| 23 | Juvenile mortality (age 3) | - 12.47 | Juvenile mortality (age 4) | - 2.59 |
| 24 | Juvenile mortality (age 4) | - 13.31 | Juvenile mortality (age 2) | - 2.63 |
| 25 | Juvenile mortality (age 2) | - 13.37 | Aquatic vegetation | - 2.71 |
| 26 | Adult mortality | - 14.10 | Juvenile mortality (age 3) | - 3.02 |
| 27 | Aquatic vegetation | - 14.74 | Carrying capacity | - 3.04 |
| 28 | Carrying capacity | - 15.03 | Adult mortality | - 3.70 |
|  |  | *N_T_* |  | *N_T_* |
| 1 | Fecundity | 6094.42 | Juvenile mortality (age 1) | 3979.91 |
| 2 | Juvenile mortality (age 1) | 4093.23 | Adult mortality | 1511.39 |
| 3 | Juvenile mortality (age 2) | 1837.35 | Juvenile mortality (age 2) | 1412.81 |
| 4 | Juvenile mortality (age 3) | 662.4 | Fecundity | 1182.49 |
| 5 | Juvenile mortality (age 4) | 248.41 | Juvenile mortality (age 3) | 444.13 |
| 6 | ES in juvenile mortality (age 1) | 128.39 | Juvenile mortality (age 4) | 274.19 |
| 7 | Aquatic vegetation | 118.87 | ES in juvenile mortality (age 1) | 223.94 |
| 8 | Adult mortality | 110.31 | ES in adult mortality | 166.94 |
| 9 | ES in juvenile mortality (age 2) | 79.25 | ES in juvenile mortality (age 2) | 105.51 |
| 10 | ES in juvenile mortality (age 3) | 48.43 | Aquatic vegetation | 93.41 |
| 11 | SD in fecundity | 26.18 | ES in juvenile mortality (age 3) | 86.59 |
| 12 | Carrying capacity | 11.57 | ES in juvenile mortality (age 1) | 63.47 |
| 13 | ES in juvenile mortality (age 4) | 10.26 | ES in juvenile mortality (age 4) | 60.51 |
| 14 | ES in adult mortality | 7.74 | Carrying capacity | 59.45 |
| 15 | ES in juvenile mortality (age 2) | -7.12 | ES in juvenile mortality (age 4) | 27.46 |
| 16 | ES in juvenile mortality (age 3) | -7.52 | SD in fecundity | 21.5 |
| 17 | ES in juvenile mortality (age 1) | -14.11 | SD in fecundity | -10.11 |
| 18 | SD in fecundity | -15.14 | ES in juvenile mortality (age 2) | -24.11 |
| 19 | ES in juvenile mortality (age 4) | -18.57 | ES in adult mortality | -26.49 |
| 20 | ES in adult mortality | -194.51 | ES in juvenile mortality (age 3) | -34.65 |
| 21 | Juvenile mortality (age 3) | -249.23 | SD in fecundity | -94.31 |
| 22 | Juvenile mortality (age 1) | -250.98 | Aquatic vegetation | -116.96 |
| 23 | Fecundity | -259.06 | Juvenile mortality (age 3) | -134.03 |
| 24 | Juvenile mortality (age 2) | -269.24 | Juvenile mortality (age 4) | -138.07 |
| 25 | Aquatic vegetation | -278.01 | Carrying capacity | -138.11 |
| 26 | Juvenile mortality (age 4) | -280.14 | Juvenile mortality (age 1) | -145.23 |
| 27 | Carrying capacity | -299.01 | Juvenile mortality (age 2) | -178.23 |
| 28 | Adult mortality | -314.39 | Adult mortality | -189.26 |

**Figure S1:** Results from sensitivity analysis 2. Changes in effective population size (N_e_) with incremental changes in input parameters in the forest (green) and agricultural (purple) model.

**Figure S2:** Results from sensitivity analysis 2. Changes in total population size (N_T_) with incremental changes in input parameters in the forest (green) and agricultural (purple) model.

**Figure S3:** Results from sensitivity analysis 2. Changes in heterozygosity (H_e_) with incremental changes in input parameters in the forest (green) and agricultural (purple) model.

**Table S6:** Simulation results from the forest metapopulation scenarios. Green showing baseline scenario.

| **Rank** | **Scenario** | **H_e_** | **H_e_**  **1.96SE** | **Scenario** | **N_T_** | **N_T_**  **1.96SE** | **Scenario** | **G_ST_** |
| --- | --- | --- | --- | --- | --- | --- | --- | --- |
| 1 | JUV1 | 0.606 | 0.000 | JUV1 | 11 567 | 152 | JUV1 | 0.077 |
| 2 | JUV1ES | 0.602 | 0.000 | FEC | 7 824 | 129 | COR | 0.082 |
| 3 | FEC | 0.602 | 0.000 | JUV1ES | 5 256 | 82 | FEC | 0.086 |
| 4 | COR | 0.598 | 0.000 | BASE | 4 278 | 88 | JUV1ES | 0.088 |
| 5 | HAB | 0.597 | 0.000 | HAB | 4 194 | 84 | HAB | 0.099 |
| 6 | BASE | 0.597 | 0.000 | COR | 4 176 | 87 | BASE | 0.100 |
| 7 | DRY | 0.597 | 0.000 | 0MIG | 4 064 | 82 | DRY | 0.102 |
| 8 | 0MIG | 0.596 | 0.000 | DRY | 3 837 | 80 | TER | 0.113 |
| 9 | TER | 0.592 | 0.001 | TER | 2 803 | 65 | ADES20 | 0.133 |
| 10 | ADES20 | 0.555 | 0.002 | ADES20 | 2 195 | 69 | 0MIG | 0.135 |

**Table S7:** Simulation results from the agricultural metapopulation scenarios. Purple showing baseline scenario.

| **Rank** | **Scenario** | **H_e_** | **H_e_**  **1.96SE** | **Scenario** | **N_T_** | **N_T_**  **1.96SE** | **Scenario** | **G_ST_** |
| --- | --- | --- | --- | --- | --- | --- | --- | --- |
| 1 | ADM+ADES5 | 0.531 | 0.000 | ADM+ADES5 | 30 122 | 385 | JUV1+ADES5 | 0.012 |
| 2 | JUV1+ADES5 | 0.530 | 0.000 | JUV1+ADES5 | 28 220 | 394 | ADM+ADES5 | 0.013 |
| 3 | FEC+ADES5 | 0.527 | 0.000 | ADM | 17 438 | 354 | ADM | 0.019 |
| 4 | ADM | 0.525 | 0.000 | JUV1 | 15 917 | 342 | JUV1 | 0.020 |
| 5 | COR+ADES5 | 0.525 | 0.000 | FEC+ADES5 | 13 336 | 287 | FEC+ADES5 | 0.021 |
| 6 | ADES5 | 0.525 | 0.000 | HAB+ADES5 | 10 615 | 265 | COR+ADES5 | 0.023 |
| 7 | HAB+ADES5 | 0.524 | 0.000 | ADES5 | 9 367 | 225 | HAB+ADES5 | 0.025 |
| 8 | JUV1 | 0.522 | 0.000 | COR+ADES5 | 9 329 | 225 | ADES5 | 0.025 |
| 9 | JUV1ES | 0.520 | 0.001 | JUV1ES | 8 247 | 219 | JUV1ES | 0.027 |
| 10 | FEC | 0.510 | 0.001 | FEC | 7 014 | 218 | FEC | 0.031 |
| 11 | HAB | 0.507 | 0.001 | HAB | 5 735 | 208 | COR | 0.033 |
| 12 | COR | 0.506 | 0.001 | BASE | 5 082 | 179 | HAB | 0.035 |
| 13 | BASE | 0.506 | 0.001 | COR | 4 978 | 182 | BASE | 0.035 |
| 14 | DRY | 0.504 | 0.001 | DRY | 4 484 | 167 | DRY | 0.037 |
| 15 | TER | 0.501 | 0.001 | TER | 3 734 | 146 | TER | 0.039 |
| 16 | 0MIG | 0.493 | 0.001 | 0MIG | 3 271 | 117 | 0MIG | 0.117 |

**Table S8:** Simulation results for heterozygosity in the forest subpopulations. Green showing baseline scenario.

| **Scenario** | **Pop A** | **1.96SE** | **Scenario** | **Pop B** | **1.96SE** | **Scenario** | **Pop C** | **1.96SE** | **Scenario** | **Pop D** | **1.96SE** | **Scenario** | **Pop E** | **1.96SE** |
| --- | --- | --- | --- | --- | --- | --- | --- | --- | --- | --- | --- | --- | --- | --- |
| JUV1 | 0.557 | 0.001 | JUV1 | 0.550 | 0.000 | JUV1 | 0.579 | 0.001 | JUV1 | 0.561 | 0.001 | JUV1 | 0.566 | 0.001 |
| COR | 0.554 | 0.001 | JUV1ES | 0.548 | 0.000 | JUV1ES | 0.572 | 0.001 | COR | 0.550 | 0.002 | JUV1ES | 0.557 | 0.001 |
| FEC | 0.551 | 0.001 | FEC | 0.548 | 0.001 | FEC | 0.570 | 0.001 | FEC | 0.547 | 0.001 | FEC | 0.555 | 0.001 |
| JUV1ES | 0.550 | 0.001 | COR | 0.547 | 0.001 | COR | 0.567 | 0.001 | JUV1ES | 0.542 | 0.001 | COR | 0.548 | 0.002 |
| HAB | 0.534 | 0.001 | BASE | 0.540 | 0.001 | HAB | 0.552 | 0.002 | HAB | 0.513 | 0.002 | HAB | 0.535 | 0.002 |
| DRY | 0.533 | 0.001 | HAB | 0.540 | 0.001 | BASE | 0.551 | 0.002 | BASE | 0.511 | 0.002 | BASE | 0.531 | 0.002 |
| BASE | 0.533 | 0.002 | DRY | 0.538 | 0.001 | DRY | 0.549 | 0.002 | DRY | 0.506 | 0.002 | DRY | 0.531 | 0.002 |
| TER | 0.514 | 0.002 | 0MIG | 0.529 | 0.001 | TER | 0.527 | 0.002 | TER | 0.473 | 0.003 | TER | 0.503 | 0.003 |
| 0MIG | 0.488 | 0.002 | TER | 0.528 | 0.001 | 0MIG | 0.451 | 0.003 | ADES20 | 0.367 | 0.004 | 0MIG | 0.502 | 0.002 |
| ADES20 | 0.414 | 0.004 | ADES20 | 0.438 | 0.004 | ADES20 | 0.417 | 0.004 | 0MIG | 0.336 | 0.004 | ADES20 | 0.386 | 0.004 |
|  |  |  |  |  |  |  |  |  |  |  |  |  |  |  |
| **Scenario** | **Pop G** | **1.96SE** | **Scenario** | **Pop H** | **1.96SE** | **Scenario** | **Pop I** | **1.96SE** | **Scenario** | **Pop J** | **1.96SE** | **Scenario** | **Pop M** | **1.96SE** |
| JUV1 | 0.577 | 0.001 | COR | 0.531 | 0.001 | COR | 0.441 | 0.002 | JUV1 | 0.544 | 0.001 | JUV1 | 0.536 | 0.001 |
| FEC | 0.554 | 0.002 | JUV1 | 0.528 | 0.000 | JUV1 | 0.439 | 0.001 | JUV1ES | 0.526 | 0.002 | JUV1ES | 0.522 | 0.001 |
| COR | 0.548 | 0.002 | FEC | 0.524 | 0.001 | FEC | 0.429 | 0.001 | FEC | 0.524 | 0.002 | FEC | 0.520 | 0.002 |
| JUV1ES | 0.544 | 0.002 | JUV1ES | 0.522 | 0.001 | JUV1ES | 0.427 | 0.001 | COR | 0.515 | 0.002 | COR | 0.513 | 0.002 |
| HAB | 0.502 | 0.003 | HAB | 0.512 | 0.001 | HAB | 0.407 | 0.002 | HAB | 0.490 | 0.003 | HAB | 0.490 | 0.002 |
| BASE | 0.502 | 0.003 | BASE | 0.512 | 0.001 | BASE | 0.405 | 0.002 | BASE | 0.489 | 0.003 | BASE | 0.488 | 0.002 |
| DRY | 0.493 | 0.003 | DRY | 0.510 | 0.001 | DRY | 0.405 | 0.002 | DRY | 0.486 | 0.003 | DRY | 0.484 | 0.002 |
| TER | 0.449 | 0.004 | TER | 0.497 | 0.002 | TER | 0.379 | 0.003 | TER | 0.448 | 0.003 | TER | 0.452 | 0.003 |
| ADES20 | 0.344 | 0.005 | 0MIG | 0.463 | 0.002 | 0MIG | 0.367 | 0.002 | 0MIG | 0.439 | 0.003 | 0MIG | 0.451 | 0.003 |
| 0MIG | 0 | 0 | ADES20 | 0.408 | 0.004 | ADES20 | 0.278 | 0.004 | ADES20 | 0.320 | 0.005 | ADES20 | 0.329 | 0.005 |

**Table S9:** Simulation results for N_T_ for the forest subpopulations. Green showing baseline scenario.

| **Scenario** | **Pop A** | **1.96SE** | **Scenario** | **Pop B** | **1.96SE** | **Scenario** | **Pop C** | **1.96SE** | **Scenario** | **Pop D** | **1.96SE** | **Scenario** | **Pop E** | **1.96SE** |
| --- | --- | --- | --- | --- | --- | --- | --- | --- | --- | --- | --- | --- | --- | --- |
| JUV1 | 1 336 | 22 | JUV1 | 3 465 | 58 | JUV1 | 1 397 | 27 | JUV1 | 383 | 7 | JUV1 | 955 | 16 |
| FEC | 915 | 20 | FEC | 2 390 | 52 | FEC | 924 | 23 | FEC | 255 | 6 | FEC | 660 | 15 |
| JUV1ES | 632 | 13 | JUV1ES | 1 679 | 34 | JUV1ES | 558 | 14 | COR | 161 | 4 | JUV1ES | 469 | 9 |
| COR | 518 | 14 | 0MIG | 1 411 | 38 | COR | 487 | 15 | JUV1ES | 158 | 4 | BASE | 370 | 10 |
| BASE | 510 | 14 | BASE | 1 373 | 37 | BASE | 465 | 15 | BASE | 131 | 4 | HAB | 368 | 10 |
| HAB | 506 | 14 | HAB | 1 336 | 36 | HAB | 463 | 15 | HAB | 128 | 4 | 0MIG | 362 | 10 |
| DRY | 466 | 13 | COR | 1 250 | 34 | DRY | 414 | 14 | DRY | 116 | 4 | COR | 353 | 10 |
| 0MIG | 458 | 13 | DRY | 1 233 | 34 | 0MIG | 370 | 14 | 0MIG | 89 | 4 | DRY | 335 | 9 |
| TER | 338 | 11 | TER | 901 | 29 | TER | 300 | 11 | TER | 85 | 3 | TER | 248 | 8 |
| ADES20 | 261 | 12 | ADES20 | 672 | 31 | ADES20 | 252 | 13 | ADES20 | 69 | 3 | ADES20 | 187 | 9 |
|  |  |  |  |  |  |  |  |  |  |  |  |  |  |  |
| **Scenario** | **Pop G** | **1.96SE** | **Scenario** | **Pop H** | **1.96SE** | **Scenario** | **Pop I** | **1.96SE** | **Scenario** | **Pop J** | **1.96SE** | **Scenario** | **Pop M** | **1.96SE** |
| JUV1 | 41 | 1 | JUV1 | 1 515 | 28 | JUV1 | 952 | 19 | JUV1 | 728 | 17 | JUV1 | 794 | 16 |
| COR | 36 | 1 | FEC | 1 036 | 24 | FEC | 630 | 16 | FEC | 464 | 13 | FEC | 523 | 13 |
| FEC | 28 | 0 | JUV1ES | 694 | 15 | JUV1ES | 421 | 10 | JUV1ES | 288 | 8 | JUV1ES | 339 | 8 |
| JUV1ES | 19 | 0 | BASE | 566 | 17 | BASE | 335 | 11 | COR | 236 | 9 | BASE | 278 | 9 |
| BASE | 16 | 0 | HAB | 554 | 16 | 0MIG | 328 | 11 | BASE | 234 | 9 | 0MIG | 273 | 9 |
| HAB | 16 | 0 | 0MIG | 552 | 17 | COR | 326 | 10 | HAB | 234 | 8 | COR | 272 | 9 |
| DRY | 14 | 0 | COR | 537 | 15 | HAB | 325 | 11 | 0MIG | 221 | 8 | HAB | 264 | 8 |
| TER | 10 | 0 | DRY | 504 | 15 | DRY | 300 | 10 | DRY | 213 | 8 | DRY | 242 | 8 |
| ADES20 | 8 | 0 | TER | 372 | 13 | TER | 218 | 9 | TER | 151 | 6 | TER | 179 | 7 |
| 0MIG | 0 | 0 | ADES20 | 293 | 14 | ADES20 | 181 | 9 | ADES20 | 125 | 8 | ADES20 | 147 | 8 |

**Table S10:** Simulation results for He for the agricultural subpopulations. Purple showing baseline scenario.

| **Scenario** | **Pop 76** | **1.96SE** | **Scenario** | **Pop 94** | **1.96SE** | **Scenario** | **Pop 95** | **1.96SE** | **Scenario** | **Pop 127** | **1.96SE** | **Scenario** | **Pop 128** | **1.96SE** |
| --- | --- | --- | --- | --- | --- | --- | --- | --- | --- | --- | --- | --- | --- | --- |
| JUV1+ADES5 | 0.521 | 0.000 | ADM+ADES5 | 0.453 | 0.000 | JUV1+ADES5 | 0.509 | 0.000 | JUV1+ADES5 | 0.514 | 0.000 | ADM+ADES5 | 0.515 | 0.000 |
| ADM+ADES5 | 0.520 | 0.000 | JUV1+ADES5 | 0.451 | 0.001 | ADM+ADES5 | 0.508 | 0.000 | ADM+ADES5 | 0.514 | 0.000 | JUV1+ADES5 | 0.511 | 0.001 |
| FEC+ADES5 | 0.516 | 0.000 | COR+ADES5 | 0.445 | 0.001 | ADM | 0.503 | 0.001 | ADM | 0.506 | 0.001 | ADM | 0.499 | 0.001 |
| ADM | 0.515 | 0.000 | ADM | 0.442 | 0.001 | JUV1 | 0.501 | 0.001 | FEC+ADES5 | 0.504 | 0.001 | FEC+ADES5 | 0.496 | 0.001 |
| JUV1 | 0.514 | 0.001 | JUV1 | 0.440 | 0.001 | FEC+ADES5 | 0.500 | 0.001 | JUV1 | 0.503 | 0.001 | JUV1 | 0.494 | 0.001 |
| COR+ADES5 | 0.513 | 0.001 | FEC+ADES5 | 0.439 | 0.001 | COR+ADES5 | 0.499 | 0.001 | COR+ADES5 | 0.502 | 0.001 | COR+ADES5 | 0.493 | 0.001 |
| HAB+ADES5 | 0.512 | 0.000 | HAB+ADES5 | 0.434 | 0.001 | ADES5 | 0.496 | 0.001 | ADES5 | 0.499 | 0.001 | HAB+ADES5 | 0.489 | 0.001 |
| ADES5 | 0.512 | 0.001 | ADES5 | 0.433 | 0.001 | HAB+ADES5 | 0.495 | 0.001 | HAB+ADES5 | 0.498 | 0.001 | ADES5 | 0.489 | 0.001 |
| JUV1ES | 0.508 | 0.001 | COR | 0.431 | 0.002 | JUV1ES | 0.492 | 0.001 | JUV1ES | 0.495 | 0.001 | JUV1ES | 0.485 | 0.001 |
| FEC | 0.499 | 0.001 | JUV1ES | 0.430 | 0.001 | FEC | 0.487 | 0.001 | FEC | 0.489 | 0.001 | FEC | 0.477 | 0.001 |
| HAB | 0.495 | 0.001 | FEC | 0.424 | 0.001 | COR | 0.482 | 0.001 | COR | 0.483 | 0.001 | COR | 0.474 | 0.001 |
| BASE | 0.495 | 0.001 | HAB | 0.421 | 0.002 | BASE | 0.481 | 0.001 | HAB | 0.482 | 0.001 | HAB | 0.472 | 0.001 |
| COR | 0.494 | 0.001 | BASE | 0.420 | 0.002 | HAB | 0.480 | 0.001 | BASE | 0.481 | 0.001 | BASE | 0.470 | 0.001 |
| DRY | 0.493 | 0.001 | DRY | 0.417 | 0.002 | DRY | 0.478 | 0.001 | DRY | 0.480 | 0.001 | DRY | 0.468 | 0.002 |
| TER | 0.489 | 0.001 | TER | 0.416 | 0.002 | TER | 0.475 | 0.001 | TER | 0.475 | 0.001 | TER | 0.465 | 0.002 |
| 0MIG | 0.447 | 0.002 | 0MIG | 0.392 | 0.002 | 0MIG | 0.434 | 0.002 | 0MIG | 0.436 | 0.002 | 0MIG | 0.435 | 0.003 |
|  |  |  |  |  |  |  |  |  |  |  |  |  |  |  |
| **Scenario** | **Pop 138** | **1.96SE** | **Scenario** | **Pop 139** | **1.96SE** | **Scenario** | **Pop 487** | **1.96SE** | **Scenario** | **Pop 488** | **1.96SE** | **Scenario** | **Pop 494** | **1.96SE** |
| ADM+ADES5 | 0.523 | 0.000 | ADM+ADES5 | 0.526 | 0.000 | ADM+ADES5 | 0.534 | 0.000 | ADM+ADES5 | 0.537 | 0.000 | ADM+ADES5 | 0.524 | 0.000 |
| JUV1+ADES5 | 0.523 | 0.000 | JUV1+ADES5 | 0.524 | 0.000 | JUV1+ADES5 | 0.530 | 0.000 | JUV1+ADES5 | 0.534 | 0.000 | JUV1+ADES5 | 0.522 | 0.000 |
| FEC+ADES5 | 0.517 | 0.000 | FEC+ADES5 | 0.516 | 0.001 | ADM | 0.520 | 0.001 | FEC+ADES5 | 0.523 | 0.001 | FEC+ADES5 | 0.507 | 0.001 |
| ADM | 0.516 | 0.000 | ADM | 0.515 | 0.001 | FEC+ADES5 | 0.520 | 0.001 | ADM | 0.522 | 0.001 | ADM | 0.511 | 0.001 |
| COR+ADES5 | 0.515 | 0.001 | COR+ADES5 | 0.515 | 0.001 | JUV1 | 0.515 | 0.001 | COR+ADES5 | 0.520 | 0.001 | COR+ADES5 | 0.513 | 0.001 |
| JUV1 | 0.513 | 0.001 | JUV1 | 0.512 | 0.001 | COR+ADES5 | 0.515 | 0.001 | ADES5 | 0.519 | 0.001 | ADES5 | 0.500 | 0.001 |
| ADES5 | 0.513 | 0.001 | ADES5 | 0.512 | 0.001 | HAB+ADES5 | 0.514 | 0.001 | HAB+ADES5 | 0.518 | 0.001 | HAB+ADES5 | 0.501 | 0.001 |
| HAB+ADES5 | 0.513 | 0.001 | HAB+ADES5 | 0.512 | 0.001 | ADES5 | 0.514 | 0.001 | JUV1 | 0.517 | 0.001 | JUV1 | 0.506 | 0.001 |
| JUV1ES | 0.508 | 0.001 | JUV1ES | 0.506 | 0.001 | JUV1ES | 0.507 | 0.001 | JUV1ES | 0.511 | 0.001 | JUV1ES | 0.496 | 0.001 |
| FEC | 0.500 | 0.001 | FEC | 0.497 | 0.001 | FEC | 0.499 | 0.001 | FEC | 0.501 | 0.001 | FEC | 0.489 | 0.001 |
| COR | 0.496 | 0.001 | COR | 0.495 | 0.001 | BASE | 0.493 | 0.001 | COR | 0.496 | 0.001 | HAB | 0.483 | 0.001 |
| HAB | 0.495 | 0.001 | HAB | 0.493 | 0.001 | HAB | 0.493 | 0.001 | BASE | 0.495 | 0.001 | COR | 0.494 | 0.001 |
| BASE | 0.495 | 0.001 | BASE | 0.492 | 0.001 | COR | 0.493 | 0.001 | HAB | 0.495 | 0.001 | BASE | 0.482 | 0.001 |
| DRY | 0.493 | 0.001 | DRY | 0.490 | 0.001 | DRY | 0.490 | 0.001 | DRY | 0.493 | 0.001 | DRY | 0.480 | 0.001 |
| TER | 0.490 | 0.001 | TER | 0.487 | 0.001 | TER | 0.487 | 0.001 | TER | 0.489 | 0.001 | TER | 0.476 | 0.001 |
| 0MIG | 0.401 | 0.003 | 0MIG | 0.452 | 0.002 | 0MIG | 0.443 | 0.003 | 0MIG | 0.475 | 0.002 | 0MIG | 0.273 | 0.012 |

**Table S11:** Simulation results for N_T_ for the agricultural subpopulations. Purple showing baseline scenario.

| **Scenario** | **Pop 76** | **1.96SE** | **Scenario** | **Pop 94** | **1.96SE** | **Scenario** | **Pop 95** | **1.96SE** | **Scenario** | **Pop 127** | **1.96SE** | **Scenario** | **Pop 128** | **1.96SE** |
| --- | --- | --- | --- | --- | --- | --- | --- | --- | --- | --- | --- | --- | --- | --- |
| ADM+ADES5 | 5 205 | 80 | ADM+ADES5 | 1 989 | 38 | ADM+ADES5 | 3 699 | 55 | ADM+ADES5 | 2 043 | 29 | ADM+ADES5 | 1 500 | 27 |
| JUV1+ADES5 | 4 829 | 81 | JUV1+ADES5 | 1 793 | 38 | JUV1+ADES5 | 3 453 | 56 | JUV1+ADES5 | 1 947 | 29 | JUV1+ADES5 | 1 397 | 27 |
| ADM | 3 003 | 75 | ADM | 1 057 | 34 | ADM | 2 084 | 51 | HAB+ADES5 | 1 243 | 39 | ADM | 835 | 24 |
| JUV1 | 2 714 | 72 | JUV1 | 938 | 32 | JUV1 | 1 900 | 49 | ADM | 1 169 | 28 | JUV1 | 759 | 23 |
| FEC+ADES5 | 2 304 | 61 | FEC+ADES5 | 828 | 26 | FEC+ADES5 | 1 776 | 45 | JUV1 | 1 099 | 27 | FEC+ADES5 | 635 | 19 |
| HAB+ADES5 | 1 877 | 61 | COR+ADES5 | 619 | 21 | HAB+ADES5 | 1 601 | 50 | FEC+ADES5 | 1 045 | 25 | HAB+ADES5 | 544 | 21 |
| ADES5 | 1 656 | 51 | HAB+ADES5 | 571 | 21 | ADES5 | 1 327 | 38 | ADES5 | 790 | 21 | COR+ADES5 | 543 | 17 |
| COR+ADES5 | 1 562 | 49 | ADES5 | 566 | 21 | COR+ADES5 | 1 307 | 37 | COR+ADES5 | 745 | 20 | ADES5 | 453 | 16 |
| JUV1ES | 1 497 | 51 | JUV1ES | 463 | 18 | JUV1ES | 1 164 | 37 | JUV1ES | 702 | 21 | JUV1ES | 393 | 15 |
| FEC | 1 220 | 49 | FEC | 409 | 21 | FEC | 903 | 35 | HAB | 638 | 31 | FEC | 321 | 15 |
| HAB | 1 008 | 48 | COR | 312 | 18 | HAB | 799 | 39 | FEC | 508 | 19 | HAB | 289 | 17 |
| BASE | 911 | 42 | BASE | 287 | 17 | BASE | 693 | 31 | BASE | 392 | 16 | COR | 287 | 14 |
| COR | 841 | 40 | HAB | 280 | 17 | COR | 648 | 29 | COR | 369 | 16 | BASE | 236 | 13 |
| DRY | 766 | 37 | DRY | 246 | 15 | DRY | 598 | 28 | DRY | 360 | 16 | DRY | 195 | 11 |
| 0MIG | 743 | 40 | 0MIG | 244 | 16 | 0MIG | 522 | 27 | TER | 311 | 15 | TER | 169 | 10 |
| TER | 665 | 36 | TER | 212 | 14 | TER | 512 | 26 | 0MIG | 286 | 15 | 0MIG | 136 | 10 |
|  |  |  |  |  |  |  |  |  |  |  |  |  |  |  |
| **Scenario** | **Pop 138** | **1.96SE** | **Scenario** | **Pop 139** | **1.96SE** | **Scenario** | **Pop 487** | **1.96SE** | **Scenario** | **Pop 488** | **1.96SE** | **Scenario** | **Pop 494** | **1.96SE** |
| ADM+ADES5 | 5 365 | 96 | ADM+ADES5 | 6 320 | 119 | ADM+ADES5 | 1 315 | 19 | ADM+ADES5 | 2 492 | 39 | ADM+ADES5 | 194 | 3 |
| JUV1+ADES5 | 5 083 | 97 | JUV1+ADES5 | 5 853 | 118 | JUV1+ADES5 | 1 281 | 20 | JUV1+ADES5 | 2 409 | 41 | JUV1+ADES5 | 175 | 2 |
| FEC+ADES5 | 3 403 | 34 | ADM | 3 512 | 104 | ADM | 853 | 20 | ADM | 1 480 | 37 | COR+ADES5 | 136 | 3 |
| ADM | 3 319 | 96 | JUV1 | 3 206 | 99 | JUV1 | 805 | 20 | JUV1 | 1 360 | 36 | ADM | 126 | 3 |
| JUV1 | 3 024 | 90 | FEC+ADES5 | 2 535 | 81 | FEC+ADES5 | 674 | 16 | FEC+ADES5 | 1 185 | 31 | JUV1 | 111 | 2 |
| HAB+ADES5 | 1 552 | 50 | HAB+ADES5 | 1 782 | 70 | HAB+ADES5 | 490 | 12 | HAB+ADES5 | 883 | 26 | FEC+ADES5 | 92 | 2 |
| ADES5 | 1 534 | 50 | ADES5 | 1 663 | 62 | ADES5 | 474 | 12 | COR+ADES5 | 840 | 24 | COR | 80 | 3 |
| COR+ADES5 | 1 464 | 47 | COR+ADES5 | 1 624 | 61 | COR+ADES5 | 490 | 13 | ADES5 | 836 | 25 | HAB+ADES5 | 72 | 2 |
| JUV1ES | 1 348 | 49 | JUV1ES | 1 511 | 61 | JUV1ES | 401 | 11 | JUV1ES | 710 | 23 | ADES5 | 67 | 1 |
| FEC | 1 288 | 57 | FEC | 1 317 | 62 | FEC | 379 | 14 | FEC | 613 | 25 | JUV1ES | 59 | 1 |
| HAB | 913 | 45 | HAB | 988 | 57 | HAB | 281 | 11 | HAB | 494 | 23 | FEC | 54 | 2 |
| BASE | 859 | 42 | BASE | 941 | 52 | BASE | 264 | 10 | BASE | 461 | 21 | HAB | 43 | 1 |
| COR | 821 | 41 | COR | 912 | 50 | COR | 267 | 11 | COR | 441 | 20 | BASE | 40 | 1 |
| DRY | 786 | 42 | DRY | 852 | 49 | DRY | 243 | 10 | DRY | 402 | 19 | DRY | 36 | 1 |
| TER | 618 | 35 | TER | 667 | 42 | TER | 209 | 9 | TER | 339 | 17 | TER | 31 | 1 |
| 0MIG | 310 | 28 | 0MIG | 621 | 45 | 0MIG | 99 | 6 | 0MIG | 310 | 18 | 0MIG | 0 | 0 |

# References

Anantharaman, R., Hall, K., Shah, V. B., & Edelman, l. (2020). Circuitscape in Julia: High Performance Connectivity Modelling to Support Conservation Decisions. *Proceedings of the JuliaCon Conferences, 1*(1), 58.

Arntzen, J. W., & Teunis, S. F. M. (1993). A six year study on the population dynamics of the crested newt Triturus cristatus following the colonization of a newly created pond. *Herpetological Journal, 3*, 99-110.

Baker, J. M. R. (1999). Abundance and survival rates of great crested newts (Triturus cristatus) at a pond in central England: monitoring individuals. *Herpetological Journal, 9*, 1-8.

Bode, C. A., Limm, M. P., Power, M. E., & Finlay, J. C. (2014). Subcanopy Solar Radiation model: Predicting solar radiation across a heavily vegetated landscape using LiDAR and GIS solar radiation models. *Remote sensing of environment, 154*, 387-397. doi:10.1016/j.rse.2014.01.028

Boehner, J., & Selige, T. (2006). Spatial prediction of soil attributes using terrain analysis and climate regionalisation. *Goettinger Geographische Abhandlungen, 115*, 13-28.

Bülow, B. v., & Kupfer, A. (2019). Monitoring population dynamics and survival of Northern Crested Newts (Triturus cristatus) for 19 years at a pond in Central Europe. *Salamandra, 55*(2), 97–102.

Cayuela, H., Griffiths, R. A., Zakaria, N., Arntzen, J. W., Priol, P., Léna, J. P., . . . Hewison, M. (2020). Drivers of amphibian population dynamics and asynchrony at local and regional scales. *The Journal of animal ecology, 89*(6), 1350-1364. doi:10.1111/1365-2656.13208

Cayuela, H., Joly, P., Schmidt, B. R., Pichenot, J., Bonnaire, E., Priol, P., . . . Besnard, A. (2017). Life history tactics shape amphibians’ demographic responses to the North Atlantic Oscillation. *Glob Chang Biol, 23*(11), 4620-4638. doi:10.1111/gcb.13672

Cogălniceanu, D., Stănescu, F., & Arntzen, J. W. (2020). Testing the hybrid superiority hypothesis in crested and marbled newts. *Journal of zoological systematics and evolutionary research, 58*(1), 275-283. doi:10.1111/jzs.12322

Cummings, C. P., & Swan, M. J. S. (2000). Long-term survival and growth of free-living great crested newts (Triturus cristatus) pit-tagged at metamorphosis. *Herpetological Journal, 10*, 177-182.

Gallant, J. C., & Wilson, J. P. (2000). *Terrain analysis : principles and applications*. New York: Wiley.

Griffiths, R. A., Sewell, D., & McCrea, R. S. (2010). Dynamics of a declining amphibian metapopulation: Survival, dispersal and the impact of climate. *Biological Conservation, 143*, 485–491. doi:<https://doi.org/10.1016/j.biocon.2009.11.017>

Griffithsand, R. A., & Wijer, P. d. (1994). Differential effects of pH and temperature on embryonic development in the British newts (Triturus). *Journal of Zoology, 234*(4), 613-622. doi:<https://doi.org/10.1111/j.1469-7998.1994.tb04868.x>

Hagström, T. (1979). Population Ecology of Triturus cristatus and T. vulgaris (Urodela) in SW Sweden. *Holarctic Ecology, 2*(2), 108-114.

Haugen, H., Dervo, B. K., Østbye, K., Heggenes, J., Devineau, O., & Linløkken, A. (2023). Genetic diversity, gene flow, and landscape resistance in a pond-breeding amphibian in agricultural and natural forested landscapes in Norway. *Evolutionary Applications, n/a*(n/a). doi:<https://doi.org/10.1111/eva.13633>

Haugen, H., Devineau, O., Heggenes, J., Østbye, K., & Linløkken, A. (2022). Predicting Habitat Properties Using Remote Sensing Data: Soil pH and Moisture, and Ground Vegetation Cover. *Remote sensing (Basel, Switzerland), 14*(20), 5207. doi:10.3390/rs14205207

Holleley, C. E., Nichols, R. A., Whitehead, M. R., Adamack, A. T., Gunn, M. R., & Sherwin, W. B. (2014). Testing single-sample estimators of effective population size in genetically structured populations. *Conservation Genetics, 15*(1), 23-35. doi:10.1007/s10592-013-0518-3

Jarvis, L. E. (2012). *Microhabitat requirements of the great crested newt (Triturus cristatus) in a woodland area. .* (PhD Thesis), The Open University.

Miaud, C. (1994). Role of Wrapping Behavior on Egg Survival in Three Species of Triturus (Amphibia: Urodela). *Copeia, 1994*(2), 535-537. doi:10.2307/1447007

Nergaard, P. J. (2020). *Estimating survival and population size of the smooth newt and the great crested newt in a small farm pond over four years.* (Bachelor Thesis), University of Inland Norway.

Oldham, R. S. (1994). *Habitat assessment and population ecology in Conservation and management of great crested newts: Proceedings of a symposium held on 11 January 1994 at Kew Gardens, Richmond, Surrey*. Retrieved from Peterborough:

Peterman, W. E. (2018). ResistanceGA: An R package for the optimization of resistance surfaces using genetic algorithms. *Methods in Ecology and Evolution*, 1638-1647. doi:<https://doi.org/10.1111/2041-210X.12984>

Rudshaug, T. H. (2024). *Comparing count data to capture-recapture estimates, and population dynamics for two syntopic newt species, Lissotriton vulgaris and Triturus cristatus, in a rural pond.* ( Master Thesis), University of Inland Norway.

Van Buskirk, J. (2007). Body size, competitive interactions, and the local distribution of Triturus newts. *J Anim Ecol, 76*(3), 559-567. doi:10.1111/j.1365-2656.2007.01218.x
